# A polyoxyethylene sorbitan oleate modified hollow gold nanoparticle system to escape macrophage phagocytosis designed for triple combination lung cancer therapy via LDL-R mediated endocytosis

**DOI:** 10.1080/10717544.2020.1822459

**Published:** 2020-09-23

**Authors:** Yan Shen, Yun Xia, Ershuang Yang, Zixuan Ye, Yuan Ding, Jiasheng Tu, Yong Zhang, Pengcheng Xu

**Affiliations:** a Department of Pharmaceutics, Center for Research Development and Evaluation of Pharmaceutical Excipients and Generic Drugs, China Pharmaceutical University, Nanjing, China; b Department of Pharmacy, Children’s Hospital of Nanjing Medical University, Nanjing, China; c Department of Pharmaceutical Engineering, College of Pharmacy, Inner Mongolia Medical University, Hohhot, People’s Republic of China

**Keywords:** HGNPs, PSO, macrophage phagocytosis, LDL receptor, triple combination therapy

## Abstract

Presently, a combination of chemotherapy, radiotherapy, thermotherapy, and other treatments has become a hot topic of research for the treatment of cancer, especially lung cancer. In this study, novel hollow gold nanoparticles (HGNPs) were used as drug carriers, and in order to improve the targeting ability of HGNPs to a lung tumor site, polyoxyethylene sorbitol oleate (PSO) was chosen here as a target ligand since it can be specifically recognized by the low-density lipoprotein (LDL) receptor which is usually over expressed on A549 lung cancer cells. In this way, a PSO-modified doxorubicin-loaded HGNP drug delivery system (PSO-HGNPs-DOX) was constructed and its physicochemical properties, photothermal conversion ability, and drug release of PSO-HGNPs-DOX was investigated. Further, the effects of triple combination therapy, the intracellular uptake, and the ability to escape macrophage phagocytosis of PSO-HGNPs-DOX were also studied using A549 cells *in vitro*. In addition, an *in vivo* mouse model was also used to study the targeting of PSO-HGNPs-DOX to lung cancer. PSO-HGNPs-DOX demonstrated a good triple therapeutic effect for lung cancer (A549 cell viability was only 10% at 500 μM) by LDL receptor mediated endocytosis and was able to escape macrophage phagocytosis to enhance its accumulation at the target site. Therefore, PSO-HGNPs-DOX is a novel, safe, promising, and targeted drug carrier designed for triple combination lung cancer therapy which should be further studied for such applications.

## 1. Introduction

Lung cancer is a malignant tumor with high morbidity and mortality, which is a serious threat to human life and health (Chen et al., 2016). At present, the main methods for treating lung tumors include surgical treatment, chemotherapy (Mead et al., 1980), radiotherapy (Mcmahon et al., 2008), and thermotherapy. As with all cancers, early lung cancer has a higher five-year survival rate by surgical treatment. Chemotherapy and radiotherapy can improve the survival rate of advanced tumors, inhibit blood vessel growth, and impair the migration of tumor cells (Wong et al., 2012). Further, thermotherapy can improve the specific killing on tumor tissue, reduce systemic toxicity, and enhance the body’s secondary immunity to improve a patient’s quality of life (Huang et al., 2011; Jihyoun et al., 2014; Liu et al., 2017). However, there are many disadvantages to the current conventional single lung cancer treatment. For example, local recurrence and distant metastases usually occur after surgical treatment and chemotherapy (Viganò et al., 2013; Leger et al., 2017) and radiotherapy by itself has large side effects. A single thermotherapy may cause an uneven photothermal effect to the tumor site, leading to local overheating or insufficient local heating, which will not only cause tumor recurrence but also damage surrounding normal tissue (You et al., 2010). Nowadays, the use of a single lung cancer treatment strategy is inadequate to meet the growing needs for successful lung cancer treatment. To solve these problems, a combination of multiple treatment methods (such as chemotherapy, radiotherapy, and thermotherapy) into one has become a hot research topic in clinical medicine. While this smart combination strategy has demonstrated effective tumor treatment with reduced normal tissue damage, the optimal synergy of chemotherapy, thermotherapy and radiotherapy needs to be further studied for managing chemo- and radioresistant tumors. Additionally, a combination treatment of heat, drugs, and radiation will lead to reduced antitumor agent dosage and a reduction in X-rays to improve clinical therapeutic outcomes.

Gold nanoparticles (AuNPs) are promising drug delivery carriers which can also provide such a combination lung cancer therapy (Tao et al., 2008). They have many desirable properties as drug carriers, such as chemical inertness, better biocompatibility compared to other carriers, easier control of dispersion size, and so forth. Moreover, AuNPs have a high specific surface area which facilitates dense loading of targeted functional groups with therapeutic functional groups (Rana et al., 2012). Further, local surface plasmon resonance (LSPR) is a unique property of noble metal nanoparticles. When the frequency of incident light is equivalent to the vibration frequency of AuNPs, they can strongly absorb photon energy (Li et al., 2018; Wang et al., 2019). Because tissue and blood have low absorption and scattering of near-infrared light (NIR) in the 650–900 nm range, NIR in this range can penetrate nearly 10 cm without damaging healthy tissue (Pastrana, 2012); this is the depth of most lung tumors. Studies have shown that the LSPR of AuNPs has a clear relationship with the shape, size, and dispersion medium of the particles (Troutman et al., 2008). Therefore, by adjusting the LSPR of AuNPs in the near-infrared region of 650–900 nm, the absorbed light energy can be converted into heat more efficiently for photothermography or photothermal therapy (PTT). For example, when gold-nanoparticle-mediated photothermal action causes cancer cell temperatures to be higher than 42 °C, they can cause protein denaturation and rupture cancer cell membranes, which in turn causes irreversible damage to the cells (Natasha et al., 2013).

Of all of the possible AuNPs geometries, hollow gold nanoparticles (HGNPs) are the most attractive for lung cancer combination therapies since their hollow structure can form a larger absorption cross section and the LSPR can be between 650 and 900 nm by simply adjusting the thickness and size of the outer shell. HGNPs can have a higher photothermal conversion effect and are widely used for their photothermal properties for effective tumor treatment (Li et al., 2018). HGNPs can also be used as radiosensitizers and can produce strong photoelectric absorption and secondary electrons after irradiation, thus, accelerating cancer cell DNA strand breakage (Rahman et al., 2014). However, in order to obtain highly effective combination thermotherapy and radiotherapy materials that enable deep penetration into biological tissues and avoid nonspecific heating and radiation damage to normal healthy tissues, desirable photothermal agents should exhibit high specific accumulation at the tumor site and be selectively taken up by tumor cells. Therefore, the functional modification of AuNPs can increase their uptake into cancer cells to improve the heating and radiosensitization effect.

For example, Huang et al. described that a multifunctional nano-probe surface modification of folic acid (GNR-SiO_2_-FA) with silica-coated gold nanorods not only highly targets cancer cells, but also enhances radiation therapy for cancer cells (RT) and PTT effects (Huang et al., 2011). Mohamed et al. (2014) constructed a dual-targeted AuNPs system modified by folic acid and transferrin antibodies and showed that the system can effectively deliver anti-cancer drugs and combined with the photothermal ablation of AuNPs, kill tumor cells. However, there are still unresolved issues regarding the use of HGNPs as a chemotherapeutic carrier material. As a new type of inorganic nanomaterial, unmodified HGNPs can only reach tumor cells through the enhanced permeability and retention (EPR) effect because they are not targeted, which may cause certain accumulated toxicity to normal cells in the body. Presently, in most studies monoclonal antibodies are usually attached to the drug carrier so that they can react with the antigen to achieve effective targeting. Vascular endothelial factor inhibitors [such as bevacizumab and ramozumab (Gera et al., 1999)], epidermal growth factor inhibitors [such as trastuzumab and cetuximab (Paez et al., 2004)], and immune checkpoint inhibitors [such as nivolumab and pembrolizumab (Corsello et al., 2013)] are usually investigated. But, if the antibody is connected to the HGNPs by a chemical bond, the spatial structure of the antibodies changes during the reaction process and the activity of the monoclonal antibody is easily destroyed affected its stability and targeting efficiency (Yu et al., 2016). Moreover, high-strength chemical bonds also hinder the recognition process between the receptor and ligands, and the subsequent release of drugs. This treatment method is prone to drug resistance and adverse reactions. Therefore, an important question is how to improve the targeting of HGNPs to lung cancer cells in order to promote a better therapeutic effect.

A low-density lipoprotein receptor (LDL-R) is a cell surface receptor that recognizes apolipoprotein B100 and apolipoprotein E (Apo E) present in the outer phospholipid layer of LDL. Studies have shown that LDL receptors are overexpressed in some malignant tumor cells (Hu et al., 2011), especially in acute myeloid leukemia, rectal cancer, adrenal cancer, liver cancer, brain cancer, metastatic prostate cancer cells, and most importantly for here, lung cancer. These cancer cells require LDL to transport large amounts of cholesterol for cell membrane synthesis. Polysorbate 80, as a nonionic surfactant, enhances drug delivery through its coating on the surface of nanoparticles (Schwartzberg & Navari, 2018). Polysorbate 80 modified on the surface of the carrier can adsorb Apo E and Apo B to form a complex similar to LDL which is then endocytosized through the LDL receptor (Gao & Jiang, 2006; Kreuter, 2012). Previous studies in our laboratory have found that polyoxyethylene sorbitol oleate (PSO) with sorbitol as the parent structure, has better clinical safety than polysorbate 80 and that PSO can improve allergic reactions (including shock, dyspnea, hypotension, angioedema, rubella and other allergic reaction symptoms) caused by polysorbate 80 when used in injections (Li et al., 2014; Schwartzberg & Navari, 2018). It not only has the physical and chemical properties of polysorbate 80 but it also has extremely low hemolytic and sensitizing properties to be a safe and effective drug carrier with tumor targeting and obvious inhibitory effect on tumors. Therefore, PSO is a new type of nanoparticle coating that can target LDL-R and has great promise for clinical applications (Tony et al., 2007; Dreaden et al., 2012).

**Scheme 1.**
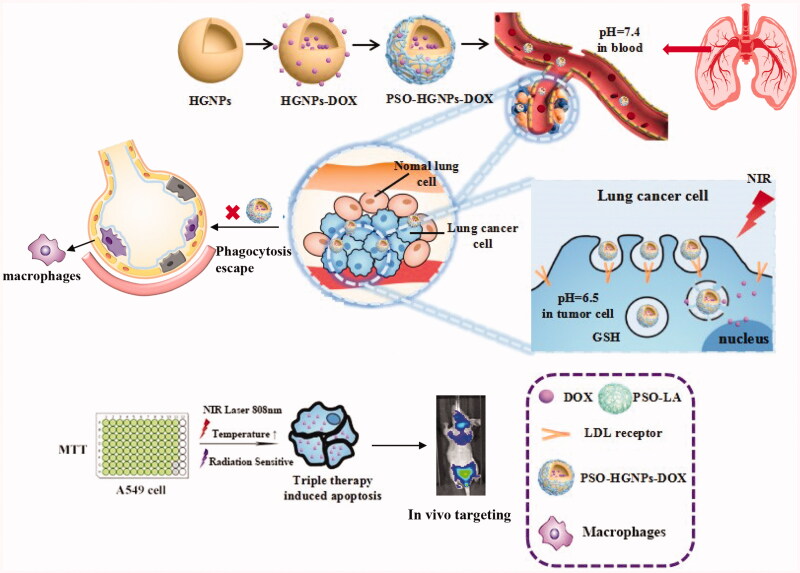
Schematic design of the present tri-therapy of a PSO modified hollow gold nanoparticles system for lung cancer targeting and treatment.

In this study, DOX was used as a model anti-cancer drug to prepare DOX-loaded HGNPs. PSO was chosen as a ligand and modified on the AuNPs here to target lung tumors for treatment via LDL receptor (Wang et al., 2020). In this way, a AuNP drug delivery system with LDL receptor targeting designed for the triple combination therapy of thermotherapy, radiotherapy, and chemotherapy was constructed in this article (Scheme 1).

## 2. Materials and methods

### 2.1. Materials and animals

PSO was purchased from Nanjing Well Chemical Co., Ltd (Nanjing, China). Doxorubicin (DOX) was obtained from Huafeng United Technology (Beijing, China). Sodium citrate (>99%), cobalt chloride hexahydrate (99.99%), sodium borohydride (99%), and chloroauric acid trihydrate (American Chemical Society reagent grade) were from Sigma Chemical Co, Ltd. (Saint Louis, MO) and were used as received. 3(4,5-dimethyl-thiazol-2-yl)-2,5-Diphenyl-tetrazolium bromide (MTT), a BCA protein concentration determination kit, a SDS-PAGE gel preparation kit and a 4′,6-diamidino-2-phenylindole (DAPI) staining kit were purchased from Jiangsu KeyGEN BioTECH Corp., Ltd (Nanjing, China). Low density lipoprotein (LDL) was bought from Aladdin (Shanghai, China) and SH-PEG-Cy7 were obtained from Xi'an Ruiqi Biotechnology Co., Ltd (Xi'an, China). All the reagents were of analytical grade and used without further purification.

A549 cells (human lung adenocarcinoma cells), L02 cells (human normal liver cells), and NR8383 cells (rat alveolar macrophage cells) were purchased from the Chinese Academy of Sciences Shanghai Cell Bank (Shanghai, China). The cells were maintained in 1640 medium containing 10% fetal calf serum purchased from Jiangsu KeyGEN BioTECH Corp., Ltd (Nanjing, China) at 37 °C in a humidified atmosphere containing 5% CO_2_. Balb/c nude mice weighing 20 ± 2 g were purchased from the Experimental Animal Center of Nanjing Qinglongshan. All animal experiments were conducted in accordance with the Guide for Laboratory Animal Facilities and Care and were approved by the Animal Ethics Committee of China Pharmaceutical University.

### 2.2. Synthesis and characterization of polyoxyethylene sorbitol oleate-lipoic acid (PSO-LA)

**Figure 1.**
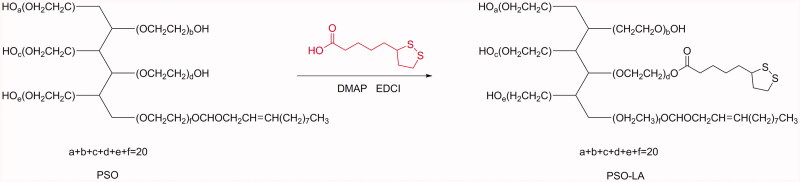
Synthesis of PSO-LA.

The synthesis procedure of PAO-LA is shown in Figure 1. The reaction was carried out at a molar ratio of lipoic acid (LA) to PSO of 2:1: 157 mg (MW 206, 0.764 mmol) of LA, 175 mg of EDC • HCl and 100 mg of 4-dimethylaminopyridine (DMAP) were weighed and dissolved in 30 mL of dimethyl sulfoxide (DMSO) for half an hour. PSO of 500 mg (Mw 1310, 0.382 mmol) was protected from light for 36 h under nitrogen. The mixture was double diluted with ultrapure water, centrifuged by a high speed refrigerated centrifuge (KDC-140HR, China) at 4000 rpm for 15 min, and the supernatant was passed through a 0.45 μm filter, dialyzed using a 1 KD dialysis bag (Shanghai yuanye Bio-Technology Co., Ltd, China), and lyophilized to obtain the product PSO-LA. The mass of the product was weighed and to provide the yield.

Oleate-LA, PSO, and PSO-LA were weighed separately, and the infrared spectrum was recorded by the KBr tablet method. The infrared spectra of LA, PSO, and PSO-LA were analyzed. DMSO-D6 was used as a solvent, the 1H-NMR spectra were recorded (300 MHz, 25 °C), and the characteristic hydrogen chemical shifts of LA, PSO, and PSO-LA were analyzed respectively.

### 2.3. Determination of LA substitution ratio on PSO-LA

The detailed information was described in the Supporting Information section.

### 2.4. Preparation and characterization of PSO-HGNPs-DOX

#### 2.4.1. Preparation of PSO-HGNPs-DOX

HGNPs and HGNPs-DOX were prepared as previously described (Li et al., 2018) and is summarized in the Supporting Information section. An appropriate amount of PSO-LA was added to the prepared HGNP-DOX solution and incubated at 37 °C for 8 h. The PSO-HGNPs-DOX solution prepared above was centrifuged at 10,000 rpm for 15 min, the unreacted PSO-LA in the supernatant was discarded, and the precipitate was re-dissolved in ultrapure water to obtain PSO-HGNPs- DOX.

#### 2.4.2. Characterization of PSO-HGNPs-DOX

The size and zeta potential of HGNP, HGNPs-DOX, PSO-HGNPs-DOX solutions were measured with particle size and zeta analyzers by Malvern Zetasizer Nano-ZS90 (Malvern instruments, UK), respectively. The morphology of HGNP, HGNPs-DOX, and PSO-HGNPs-DOX solutions were imaged using a transmission electron microscope (TEM, H-600, Hitachi, Japan) at an acceleration voltage of 200 kV. In addition, the ultra-pure water was used as the blank solution, and the plasmon resonance absorption peak and absorption spectrum of samples were determined by a UV1800 UV-Vis spectrophotometer (Shimadzu, Japan) in the wavelength range of 400 to 1000 nm.

Although the ultraviolet spectrum of PSO has only terminal absorption, the polyethoxy group in its structure can react with cobalt thiocyanate to form a blue complex. The blue complex can have a maximum absorption wavelength at 620 nm after being extracted by an organic solvent (Chun-Li et al., 2012). Therefore, in this experiment, the absorbance of PSO-HGNPs-DOX at a wavelength of 620 nm was measured by spectrophotometry, and then, the standard curve method was used to calculate the content of PSO to examine the loading of PSO on HGNPs.

At the same time, in this article, a fluorescence spectrophotometer was used to scan the DOX solution to determine the excitation wavelength and emission wavelength of the maximum absorption of DOX. Then the DOX content in the PSO-HGNPs-DOX solution was determined at DOX excitation wavelength *λ*_ex_ = 480 nm and emission wavelength *λ*_em_ = 557 nm. The absorption efficiency was calculated as the following formula:

(1) $$\text{Absorption efficiency }(\%)=\frac{m_{0}-m_{r}}{m_{0}}\times 100\%\;$$

where *m*_0_ is the weight of DOX reacted with PSO-HGNPs, *m_r_* is the weight of DOX was not absorbed onto PSO-HGNPs

### 2.5. Photothermal transformation ability of PSO-HGNPs-DOX

To verify the photothermal transduction ability of different sample solutions, HGNPs, HGNPs-DOX, and PSO-HGNPs-DOX solutions were respectively prepared at 0.075 mM, 0.15 mM, and 0.30 mM. One milliliter of each of these samples were placed in a quartz cuvette, and irradiated with a laser at 808 nm (LSR808FC, Lasever Inc, China) for 10 min at 5 W/cm^2^, and the temperature change was measured by a digital thermometer (Germany, PCETC3). The PBS solution was used as the negative control. In order to compare and optimize the photothermal effect of these samples, 0.3 mM (calculated as Au) of HGNPs, HGNPs-DOX, and PSO-HGNPs-DOX solutions were prepared and irradiated with a laser of 808 nm at 1 W/cm^2^ for 2.5 min. After the solution was cooled to room temperature, the above operation was repeated 10 times, and each temperature was recorded.

The photothermal conversion efficiency (*η*) of PSO-HGNPs-DOX samples was investigated under 808 nm laser irradiation. For thus, a PSO-HGNPs-DOX solution was diluted to 1 mg/mL, and the appropriate amount of the above solution was placed in a cuvette, the mass (m) of the sample was weighed, and the ultraviolet absorbance value (A) was then measured. Then, the solution was irradiated with an 808 nm laser at a power of 3 W/cm^2^ for 600 s and cooled naturally for 600 s. The change of temperature during the heating and cooling of PSO-HGNPs-DOX was recorded with an infrared thermal imager every 30 s. At the same time, the temperature change of ultrapure water was tested as a blank control under the same experimental conditions. The photothermal conversion efficiency (*η*) of PSO-HGNPs-DOX under 808 nm laser irradiation was calculated using formula (Abdel-Fattah, 2002; 2–6):

(2) $$\theta =\frac{T-T_{\mathrm{surr}}}{T_{\max}-T_{\mathrm{surr}}}$$

(3) $${\;\tau }_{s}=\frac{-\ln\theta }{t}$$

(4) $$hs=\frac{mc_{\mathrm{water}}}{\tau_{s}}$$

(5) $$Q_{\mathrm{dis}}=\frac{mc_{\mathrm{water}}(T_{\max\left(\mathrm{water}\right)}-T_{\mathrm{surr}})}{\tau_{\mathrm{water}}}\;$$

(6) $$\eta =\frac{hs(T_{\max}-T_{\mathrm{surr}})-Q_{\mathrm{dis}}}{I(1-10^{-A})}$$

where *h* is the heat transfer coefficient and *s* is the surface area of the container. In order to calculate *h*_s_, *θ* was introduced to define *τ_s_*. That is, *τ_s_* is the slope of –ln*θ* as the abscissa and the time of NIR irradiation as the ordinate. *T*_max_ is the highest temperature of the test sample solution, *T*_surr_ is the temperature of the surrounding environment when the sample was tested, *I* is the power density of the laser used, and *A* is the absorption intensity of the sample at 808 nm. *m* is the mass of the heated sample solution, and *C*_water_ is the specific heat capacity of water. The *Q*_dis_ in the formula is the measured value of the blank sample, where *T*_max (water)_ is the highest temperature of water heating after irradiation with NIR and *τ*_water_ is the slope between –ln*θ* of water and irradiation time.

### 2.6. *In vitro* NIR-triggered release of PSO-HGNPs-DOX

The prepared HGNPs-DOX and PSO-HGNPs-DOX solutions were centrifuged at 1000 rpm for 10 min, and the precipitate was reconstituted with ultrapure water to prepare a solution containing a DOX concentration of 8 μg/mL. Then, 2 mL of free DOX, HGNPs-DOX, PSO-HGNPs-DOX solutions were placed in separated dialysis bags (14KD) and immersed into 20 mL of release medium (PBS 6.8, PBS 7.4, PBS 6.8 + 10 mM GSH, PBS 7.4 + 10 mM GSH, respectively). One milliliter of the samples from the release medium were taken at predetermined time intervals (0.5, 1, 2, 4, 6, 8, 12, and 24 h), and the same volume of isothermal release medium was added. Also, in order to evaluate the feasibility of triggering DOX release under NIR illumination, the DOX release by 808 nm NIR irradiation was carried out at 5 W/cm^2^ for 5 min before sampling at each point. According to the fluorescence spectrophotometer, the fluorescence intensity of DOX in the sample solution at each time point was measured at the excitation wavelength *λ*_ex_ = 480 nm and the emission wavelength *λ*_em_ = 557 nm, respectively, and the concentration of DOX was calculated using a standard curve (*y* = 167.8*x* + 13.43, *R*^2^ = 0.9968). Finally, the cumulative release percentage (CRP) of the drug was calculated from the ratio of the concentration of DOX released in the supernatant before and after light irradiation to the total concentration. The CRP (%) of HGNPs-DOX and PSO-HGNPs-DOX under 808 nm laser irradiation was calculated using formula (7):

(7) $$\mathrm{CRP}=\frac{C_{n}*V_{0}+(C_{1}+C_{2}+C_{3}+\ldots +C_{n-1})*V}{Q}*100\%$$

where *C_n_*: concentration at the nth sampling point; *V*_0_: volume of release medium; and *V*: each sampling volume.

### 2.7. Triple combination therapy evaluation* in vitro*

#### 2.7.1. Cell viability after chemotherapy, thermotherapy, and radiotherapy alone on A549 cells

Cell viability after chemotherapy, thermotherapy, and radiotherapy of A549 cells were evaluated using the 3-(4,5-dimethylthiazol-2-yl)-2,5-diphenyltetrazolium bromide (MTT) colorimetric test by calculating the cell viability (%). The A549 cells in a logarithmic growth phase were seeded in a 96-well plate at a density of 5000 cells per well and were placed in a 37 °C, 5% CO_2_ cell culture incubator (Thermo Fisher Scientific, USA) for 24 h.

The detailed information of A549 cells treated with chemotherapy, thermotherapy and radiotherapy alone was described in the Supporting Information section, and the cell viability was calculated by the following formula (8):

(8) $$\text{Cell}\;\text{viability}\;=\;\left(A_{\text{sample}}-A_{\text{blank}}\right)/\left(A_{\text{control}}-A_{\text{blank}}\right)\;\times 100\%$$

where *A*_sample_ is the absorbance of cells incubated with the nanoparticle sample, *A*_blank_ is the absorbance of the PBS without cells and *A*_control_ is the absorbance of the medium with cells. The toxicity of the samples is expressed as the inhibitory concentration at which 50% of cell growth inhibition was obtained (the IC50 value).

#### 2.7.2. Triple combination therapy on A549 cells

A549 cells were seeded identically on 96-well plates as stated previously. HGNPs of 0–2000 μM, HGNPs-DOX, PSO-HGNPs, PSO-HGNPs-DOX solutions (calculated as Au) were prepared. Among them, the concentration of DOX added to HGNPs-DOX and PSO-HGNPs-DOX was 8.355 μg/mL. HGNPs, HGNPs + IR, HGNPs + NIR, HGNPs-DOX + IR + NIR groups and PSO-HGNPs, PSO-HGNPs + IR, PSO-HGNPs + NIR, and PSO-HGNPs-DOX + IR + NIR groups were set, respectively. The experimental method for the group without thermotherapy and radiotherapy and the group for thermotherapy or radiotherapy alone are consistent with the method described in Supporting Information section. For the HGNPs-DOX + IR + NIR and PSO-HGNPs-DOX + IR + NIR groups, after 12 h of culture, the old medium was discarded, 100 μL of PBS was added, and irradiated with an X-ray irradiation dose of 20 Gy. After IR, PBS was replaced with fresh medium and the cells were cultured for an additional 2 h. Then, irradiation was carried out with a NIR laser (5 W/cm^2^, 5 min). When completed, the cells were cultured for 4 h, and irradiated with an NIR laser again. After irradiation, the cells were further cultured for 6 h (for 24 h in total). After the end of the cell culture, the absorbance at 570 nm of the cells after the triple combination therapy was measured by the MTT method described in 2.7.1, and the cytotoxicity was calculated according to formula (8).

#### 2.7.3. The mechanism of radiotherapy and hyperthermia

The phosphorylatedγ-H2AX (Sigma, USA) foci is a biomarker of a DNA double-strand break. Two milliliter of different concentration of HGNPs, HGNPs-DOX, and PSO-HGNPs-DOX in PBS were added to the 6-well plate, and the X-ray irradiation dose was 20 Gy. After the irradiation, additional culturing for 4 h, the amount of hydroxyl radicals produced in the solution was determined using a hydroxyl radical test kit (A018, Nanjing Jiancheng Bioengineering Institute). The unit of hydroxyl-radical-generating ability is defined as moles of H_2_O_2_ decreased per liter in the reaction system. The ability of each sample to generate hydroxyl radicals was calculated according to formula (9).

(9) abilityhrg=ODm−ODrODs−ODb×8.824 mmol/L×1mLVS×D

the ability_hrg_ was hydroxyl-radical-generating ability, the OD_m_, OD_r_, OD_s_, and OD_b_ were the absorbances of the measured samples, controls, standards, and blanks, respectively. The concentration of the standard is 8.824 mmol/l. *V*_s_ represents the amount of sampling. *D* is the dilution factor.

In order to verify the effect of thermotherapy on the expression of HSP70 in A549 cells, A549 cells were seeded identically on 96-well plates as that stated previously and different concentrations of HGNP, HGNPs-DOX, and PSO-HGNPs-DOX solutions were added to the cells and cultured for 12 h, then the cells were irradiated with a NIR laser (as described in the 2.7.1 experimental method section), culturing for an additional 24 h, the whole protein was extracted from the cells, and the protein concentration was determined via the BCA kit method. The expression of heat shock protein HSP70 in each well was determined by Western-Blot (Bio-Rad, Power Supplies Basic, USA) while GAPDH was selected as an internal reference. The Western-Blot method used was described in our previously published article (Wang et al., 2019).

#### 2.7.4. Intracellular DOX, HGNPs-DOX, and PSO-HGNPs-DOX uptake

In order to verify the cellular uptake in different LDL-R expressed cells, A549 cells and L02 cells, the respective cells were separately seeded in 6-well plates as stated previously while different concentrations of DOX, HGNPs-DOX, and PSO-HGNPs-DOX solutions containing the same DOX concentration were added to the cells and cultured for 2, 4, and 6 h. Then, the cells were digested with trypsin, the cells were mixed evenly, centrifuged at 1000 rpm for 5 min, the supernatant discarded, an appropriate amount of PBS was added and mixed, and then analyzed by flow cytometry (*E_x_* = 488 nm; E_m_ = 570 nm).

In order to further investigate the cellular uptake of the HGNPs solution, the HGNPs-DOX solution and the PSO-HGNPs-DOX solution by A549 cells and L02 cells, A549 cells with a high expression of LDL-R and L02 cells with a low expression of LDL-R were used; detailed information for such procedure is described in the Supporting Information section. The A549 cells and L02 cells were cultured in 6-well plates at 1 × 10^5^ cells per well, and once grown to ca. 80% confluence, the cells were exposed to the HGNPs solution, the HGNPs-DOX solution, and the PSO-HGNPs-DOX solution with 0.375 mM of an Au concentration and then were incubated for 2, 4, and 6 h, respectively. Later, the cell-filled plate was placed on ice, the medium removed, and the cells were washed three times with pre-cooled PBS buffer to remove any excessive or unbound drug or nanoparticles. Five hundred microliter of the RIPA cell lysate was added to a 6-well plate, and the lysate and the cells were brought into full contact, and then repeatedly thawed at −20 °C and 4 °C. Twenty microliter of the mixture was used to detected the protein content by the BCA reagent while 1 mL of aqua regia was added to the remaining solution, digested for 2 h by a microwave digestion apparatus, and the Au content was determined using AAS (ICE-3300, Thermo Fisher Scientific, USA).

Then, the uptake ability of A549 human lung cancer cells with a high expression of LDL receptors to DOX, HGNPs-DOX, PSO-HGNPs-DOX was analyzed by laser confocal microscopy (AiryScan LSM800, Carl Zeiss AG, Germany). The A549 cells were cultured in 6-well plates and given free DOX, HGNPs-DOX, PSO-HGNPs-DOX, and then incubated for 2, 4 and 6 h, respectively. Later, the medium was removed and the cells were washed three times with PBS buffer. The cells were then stained by DAPI for 15 min, fixed with 4% paraformaldehyde, and subsequently washed twice with PBS. Finally, the A549 cells were observed under an inverted fluorescence microscope (DSZ2000, China).

#### 2.7.5. LDL-R mediated endocytosis

In order to study the mechanism of endocytosis in A549 cells, the effect of the antibody solution on the targeting activity of LDL-R was first investigated by using LDL-R saturation inhibition experiments to initially prove whether PSO-HGNPs-DOX entered cells through LDL-R-mediated endocytosis. The expression of LDL-R in different cells is mentioned in the Supporting Information. A549 cells with a high expression of the LDL receptor were used to investigate the LDL-R competition inhibition. For this, A549 cells were seeded on 6-well plates as stated previously. Solutions of HGNPs-DOX and PSO-HGNPs-DOX containing high concentrations of LDL (300 μg/mL) and low concentrations of LDL (30 μg/mL) were prepared and added to the cells separately and were cultured in a CO_2_ incubator for 4 h. Five hundred microliter of RIPA cell lysates were added to a 6-well plate and the subsequent treatment method used was as described in section 2.8.6; the protein content was measured with the BCA reagent, and the Au content per unit cell protein was determined using AAS.

#### 2.7.6. Lung macrophage escape assay *in vitro*

Macrophages (NR8383 cells) in a logarithmic growth phase were seeded in a 6- well plate at a density of 5 × 10^5^ cells/well and placed in a 37 °C cell culture incubator for 24 h. DOX, HGNPs-DOX, and PSO-HGNPs-DOX containing the same concentration (calculated as DOX) were added to the cell cultures and cultured for 0.5, 2, 4, 6, and 8 h, respectively. The cells were collected, centrifuged, washed three times with PBS and the adsorption was recorded. The fluorescent dye on the cell surface was assayed by flow cytometry (*E_x_* = 488 nm, *E_m_* = 530 nm). The samples cultured in a cell culture incubator for 4 h were centrifuged, washed with PBS, resuspended in 4% paraformaldehyde, allowed to stand at room temperature for 20 min, centrifuged and washed with PBS. The nuclei were then stained with DAPI, resuspended in 50 μL PBS, mounted with a sealer and observed under laser confocal microscopy.

### 2.8. *In vivo* targeting

Animal protocols were performed under the guidelines for the Human and Responsible Use of Animals in Research set by China Pharmaceutical University. Balb/c nude mice (20 ± 2 g) about 3–4 weeks old were purchased from Qinglongshan Farms (Nanjing, China). The animals were provided with food and water and maintained at 25 °C at a relative humidity of 55%. The tumor-bearing mice were randomly divided into three groups (*n* = 3), Balb/c nude mice were inoculated with 0.2 mL of A549 cells at a concentration of 1 × 10^5^ cells/mL under the armpit of the right upper limb, and the tumor was administered until the tumor was about 150 mm^3^. Tumor size was measured, and tumor volume was calculated as *V* = *AB*^2^/2, where *A* and *B* are the maximum and minimum diameters of the tumor, respectively. PSO-HGNPs and HGNPs (0.15 mg/mL, 20 mL) were separately incubated with SH-PEG-Cy7 (5 mg/mL, 200 μL) for 24 h at room temperature and then centrifuged at 10,000 rpm for 15 min, and the supernatant was removed to obtain Cy7-HGNPs and Cy7-HGNPs-PSO. Nude mice were injected intravenously (IV) with free Cy7, Cy7-HGNPs, and Cy7-HGNPs-PSO through the tail vein with an equivalent dose of 2 mg/kg Cy7, respectively. The *in vivo* distribution of the preparations was recorded by an IVIS Spectrum *In vivo* Imaging System (USA, PerkinElmer) equipped with an excitation band pass filter at 743 nm and an emission at 767 nm at 2, 4, and 8 h after administration.

### 2.9. Data analysis

All data are reported as mean ± SD. The results were analyzed by student’s *t*-tests (SPSS 17.0, China) with *p < .05 considered significant and **p < .01 and ***p < .001 highly significant compared to corresponding controls.

## 3. Results and discussion

### 3.1. Synthesis and characterization of PSO-LA

The structure of PSO contains active hydroxyl groups which can be esterified with the carboxyl group of LA under the action of catalysts (EDC, DMAP), thereby introducing disulfide bonds to obtain PSO-LA. The resulting product was a pale yellow viscous semi-solid soluble in water and DMSO with a yield of 86.74%.

**Figure 2.**
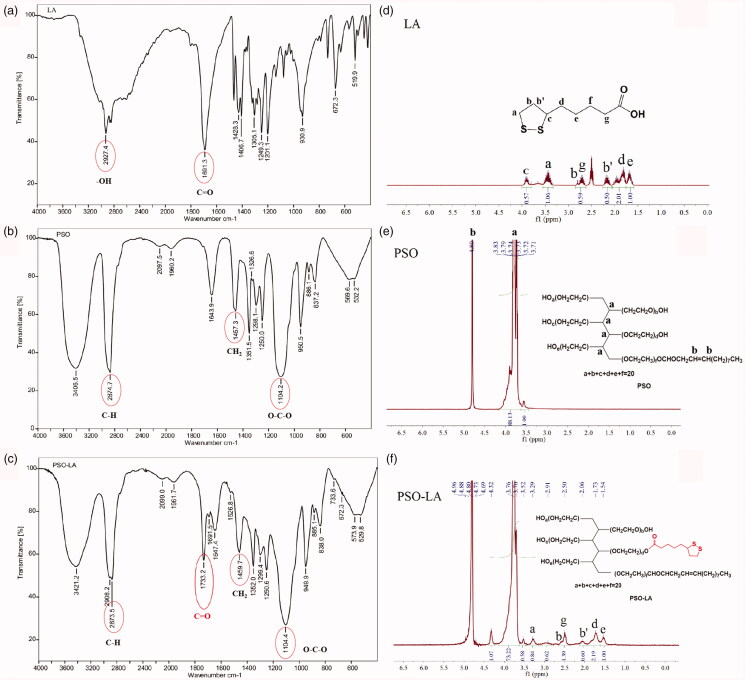
IR spectrum of LA (a), PSO (b), and PSO-LA (c). The ^1^H NMR of LA (d), PSO (e), and PSO-LA (f).

The infrared spectra of LA, PSO, and PSO-LA are shown in Figure 2(a–c). For LA, the OH– signal peak was at 2927.4 cm^−1^ and the C=O stretching vibration peak was at 1691.3 cm^−1^. For PSO, the stretching vibration peak of C–H was at 2874.7 cm^−1^, the bending vibration peak of methylene CH_2_ was at 1457.3 cm^−1^, and the antisymmetric stretching vibration of the C–O–C bond was at 1104.2 cm^−1^. It can be seen from the IR spectrum for PSO-LA that PSO–LA has the characteristic peak of PSO (CH stretching vibration peak at 2873.5 cm^−1^, bending vibration peak of methylene CH_2_ at 1459.7 cm^−1^, and the antisymmetric stretching vibration of C–O–C bond at 1104.2 cm^−1)^. In addition, PSO-LA showed a new peak at 1733.2 cm^−1^, which is the stretching vibration peak of –O–C = O, indicating that the product contained an ester bond. The above results are consistent with the structure of LA, PSO, and PSO-LA.

The ^1^H NMR spectra of LA, PSO, and PSO-LA are shown in Figure 2(d–f). It can be seen that the characteristic peak of LA appears at 1.5–4.0 ppm [(a) −CH_2_−S, 3.5 ppm; (b) and (g) −CH_2_−, 2.5 ppm; (c) −CH–S–, 3.80 ppm]. The characteristic peaks of PSO were evident [(a) −CH−, 3.71 ppm; (b) −CH = CH−, 4.80 ppm]. Compared with LA and PSO, PSO-LA showed both characteristic peaks of LA and characteristic peaks of PSO at 1.5–5.0 ppm, as indicated by ^1^H NMR. Characteristic peaks also appeared at 4.8, 3.7, 3.5, 3.29, and 2.5 ppm, respectively, which proved that the synthesis of PSO-LA was successful. The above results were consistent with the structure of LA, PSO, and PSO-LA.

### 3.2. Determination of the degree of LA substitution

The mass percentages of C, H, and S in PSO and PSO-LA at different molar ratios were obtained. The results from elemental analysis showed that PSO did not contain S, which was consistent with its structural formula. When the molar ratio of LA to PSO was 1.5:1, the degree of substitution of LA in PSO-LA was 1.30 (1.30 LAs per PSO on average). When the molar ratio of LA to PSO was 2:1, the calculated degree of substitution of LA in PSO-LA was 2.04. In addition, when the molar ratio of LA and PSO was 5:1, the obtained product was a solid substance with extremely poor water solubility.

The Ellman’s reagent method was used to determine the degree of substitution of LA in PSO-LA. The thiol concentration showed a good linear relationship with the absorbance value within 0–2 mM. In this experiment, the S-S of LA was broken by DTT to expose the -SH, and the content of -SH was measured by the Ellman reagent. When the molar ratio of LA to PSO was 1.5:1 and 2:1, the degree of substitution of LA in PSO-LA was 1.04 and 1.64, respectively. The degree of substitution of LA measured by the Ellman reagent method was smaller than that determined by elemental analysis. It was speculated that the S-S in PSO-LA cannot be completely disconnected under the DTT condition. In addition, the newly disconnected S-S can also be oxidized to form S-S again, so the results obtained by elemental analysis are considered more accurate.

In summary, a molar ratio of LA to PSO of 2:1 was selected for the subsequent experiments. This product had good water solubility and a high degree of substitution (2.04) and was easy to form PSO-HGNPs by connecting HGNPs and PSO-LA via Au-S bonds.

### 3.3. Characterization of PSO-HGNPs-DOX

PSO-HGNPs-DOX was prepared by binding PSO to the surface of HGNPs-DOX via Au-S covalent bonds. The prepared PSO-HGNPs-DOX solution was transparent and clear gray-green with a uniform appearance.

**Figure 3.**
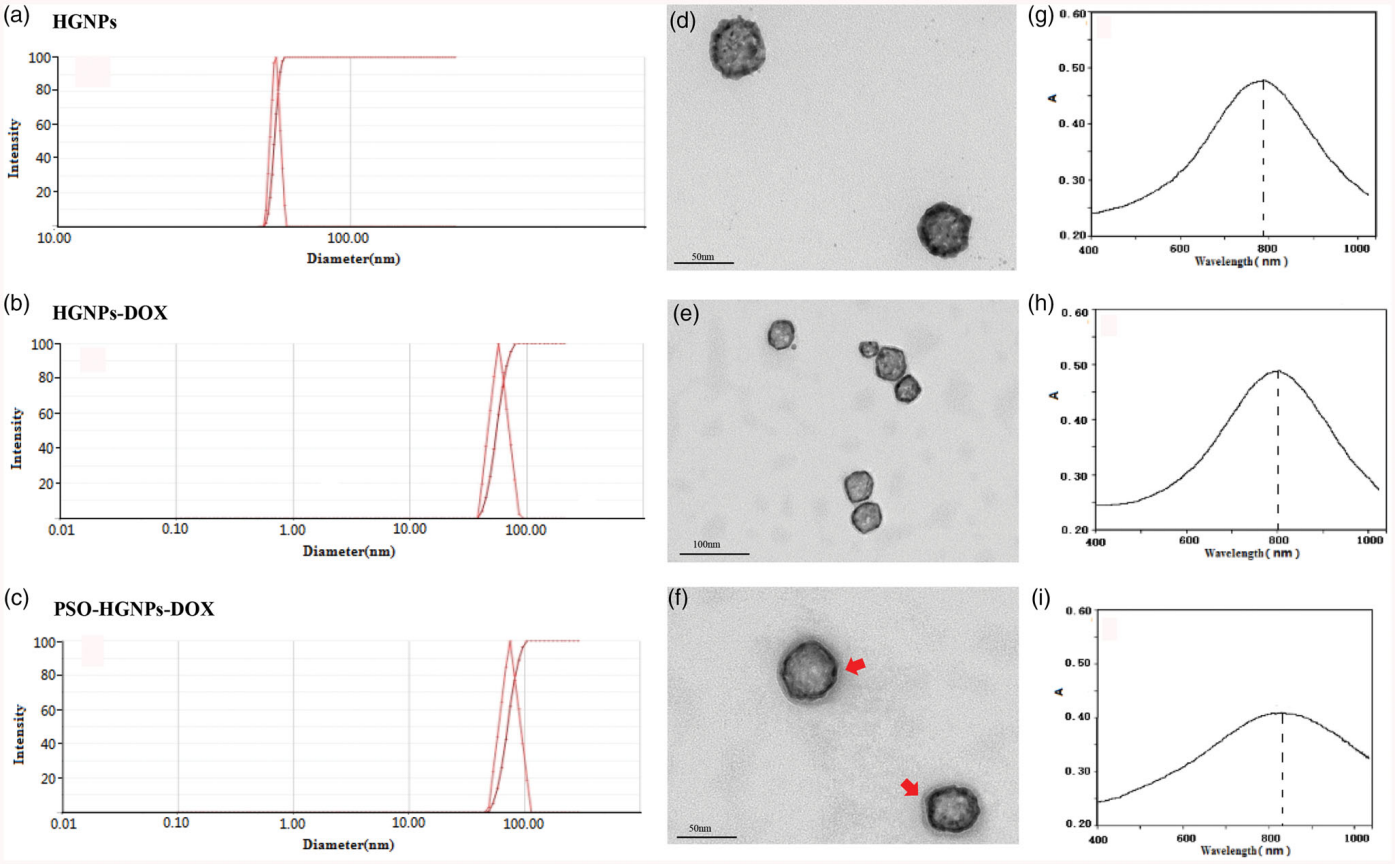
The size distribution of HGNPs (a), HGNPs-DOX (b), PSO-HGNPs-DOX (c). The TEM image of HGNPs (d), HGNPs-DOX (e), PSO-HGNPs-DOX (red arrow represents the hydration layer of PSO) (f), and the absorption spectra of HGNPs (g), HGNPs-DOX (h), and PSO-HGNPs-DOX (i).

As shown in Figure 3(a–c), the particle size of HGNPs was 53.00 ± 0.196 nm, and the zeta potential was −55.44 ± 3.88 mV. Compared with HGNPs, the particle size of HGNPs-DOX increased to 72.60 ± 0.121 nm, and the zeta potential was −39.51 ± 3.62 mV. While the particle size of PSO-HGNPs-DOX increased significantly to 99.39 ± 0.169 nm, and the zeta potential was −20.24 ± 3.72 mV. The morphological images of HGNPs, HGNPs-DOX, and PSO-HGNPs-DOX under TEM are shown in Figure 3(d–f). It can be seen that the prepared HGNPs were spherical and had a cavity structure with a particle size of about 50 nm and a shell thickness of 4–6 nm, which was consistent with the measured particle size. Compared with HGNPs, HGNPs-DOX, and PSO-HGNPs-DOX still had a spherical structure, but HGNPs-DOX and PSO-HGNPs-DOX had a gray-black membranous substance with a shell thickness of about 5 nm, which was presumed to be a hydration layer of PSO as marked by a red arrow in Figure 3(f). The TEM results confirmed that the synthesized AuNPs were spherical with a hollow structure, and it was confirmed that other substances were modified on the outside, which should be DOX and PSO. As shown in Figure 3(g–i), the maximum absorption wavelengths of the plasmon resonance absorption (SPR) peaks of the prepared HGNPs, HGNPs-DOX, and PSO-HGNPs-DOX were 785 nm, 800 nm, and 830 nm, respectively. The experimental results showed that the maximum absorption wavelengths of the SPR absorption peaks of HGNPs, HGNPs-DOX, and PSO-HGNPs-DOX were red-shifted, which were often related to changes in the introduction of chromophores, solvent polarity, or steric hindrance. When a conjugate system was introduced into the structure, the absorption band will be redshifted. As the steric hindrance of HGNPs, HGNPs-DOX, and PSO-HGNPs-DOX increased in sequence, the ultraviolet absorption wavelengths were sequentially red-shifted.

The experimental results show that PSO successfully binds to the surface of HGNPs-DOX via Au-S covalent bonds, and PSO-HGNPs-DOX is obtained. The standard curve of PSO was *y* = 0.0045*x* + 0.0182 (*R*^2^ = 0.9978). The loading rate of PSO in PSO-HGNPs-DOX was 43.29%. In addition, the drug DOX was also successfully loaded on the surface of HGNPs by physical adsorption, and PSO-HGNPs-DOX was prepared. According to the standard curve *y* = 167.8*x* + 13.43 (*R*^2^ = 0.9968), the adsorption efficiency of DOX was 82.97%. That is, the mass ratio of HGNPs (in Au) to DOX in PSO-HGNPs-DOX is 15: 8. Furthermore, the detailed information of Stability of PSO-HGNPs-DOX was shown in Supporting Information Figure S1.

### 3.4. *In vitro* photothermal transformation ability of PSO-HGNPs-DOX

With the absorption of near-infrared spectroscopy, HGNPs can exert a photothermal conversion ability with the temperature of the cancer area rising rapidly inducing the release of DOX, killing cancer cells together with thermotherapy and chemotherapy. In order to investigate the photothermal conversion capabilities of HGNPs, HGNPs-DOX, and PSO-HGNPs-DOX, continuous laser irradiation (5 W/cm^2^, 10 min) and pulsed laser (5 W/cm^2^, 5 min/cycle, 10 cycles) were studied.

**Figure 4.**
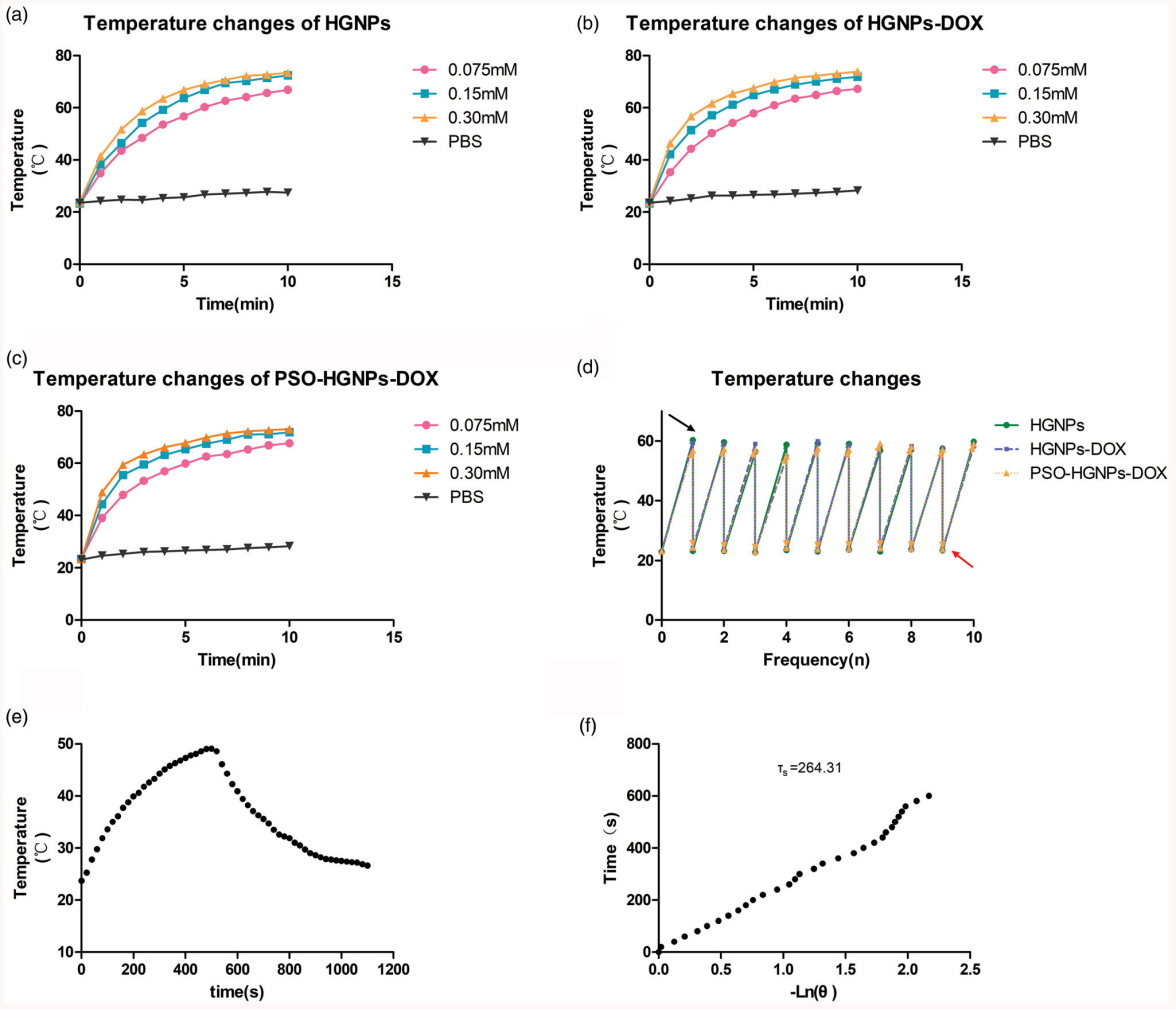
Temperature changes of HGNPs (a), HGNPs-DOX (b), and PSO-HGNPs-DOX (c) at different concentrations after continuous NIR irradiation. Temperature changes of HGNPs, HGNPs-DOX, and PSO-HGNPs-DOX after 10 repeated irradiations (red arrow indicates temperature before illumination and black arrow indicates temperature after illumination) (d). Photothermal properties of the PSO-HGNPs-DOX solution at 808 nm (5 W/cm^2^), then the irradiation lasted for 600 s and was then shut off (e). Plot of the cooling time versus –ln*θ* from the cooling stage (f).

As shown in Figure 4(a–c), different concentrations of HGNPs, HGNPs-DOX, and PSO-HGNPs-DOX solutions were irradiated with NIR laser (5 W/cm^2^, 10 min), and temperature changes were monitored. Taking the high concentration (0.3 mM) temperature increase curve as an example, the results showed there was a steep temperature increase over the first 2 min for HGNP, HGNP-DOX, and PSO-HGNP-DOX, and the temperature reached above 60 °C during 10 min. The temperature increase of HGNPs (Δ*T* of ca. 49.5 °C), HGNPs-DOX (Δ*T* of ca. 50 °C), and PSO-HGNPs-DOX (Δ*T* of ca. 49.7 °C) solutions during 10 min were significantly higher compared to PBS (Δ*T* of ca. 5.2 °C) under the same laser irradiation conditions. However, there was no significant difference in temperature increase of HGNP, HGNP-DOX, and PSO-HGNP-DOX solutions at the same concentration for the same time, indicating that the photothermal conversion capabilities of the three are almost comparable, and proving that drug loading and PSO modification did not have any significant impact on photothermal conversion ability. Moreover, an obvious concentration-dependent temperature increase was observed in Figure 4, where 60 °C was achieved within 7 min at relatively low concentration levels (0.075 mM) and was achieved within 5 min at relatively high concentration levels (0.3 mM), which proved that the concentration of gold was the main factor affecting the photothermal conversion ability of HGNPs, HGNPs-DOX, and PSO-HGNPs-DOX. This can be explained by the large cross section of absorption attributed to the cavity structure of the HGNPs (Schwartzberg et al., 2006).

As shown in Figure 4(d), 0.15 mM of the HGNPs, HGNPs-DOX, and PSO-HGNPs-DOX solutions were irradiated with an NIR laser (5 W/cm^2^, 5 min/cycle, 10cycles) and the temperature of the samples were recorded. The results showed that the maximum temperature of the HGNPs, HGNPs-DOX, and PSO-HGNPs-DOX solutions to around 60 °C was reached 5 min after each irradiation, which suggested that the photothermal conversion capacity of AuNPs would not decrease after multiple irradiations, and the light-to-heat conversion efficiency did not decrease as well.

As shown in Figure 4(e,f), the *τ_s_* of the blank control water was 345.87, and the specific heat capacity of the water was 4.2 J/(kg °C while the *τ_s_* of the PSO-HGNPs-DOX was 264.31. According to the formula, the photothermal conversion efficiency (*η*) of the PSO-HGNPs-DOX sample under the NIR of 808 nm laser (5 W/cm^2^) can be calculated as 22.43%. As an inorganic photothermal material, PSO-HGNPs-DOX has high photothermal conversion efficiency in red light, and it is a potential photosensitizer with photothermal treatment.

### 3.5. *In vitro* release of PSO-HGNPs-DOX

**Figure 5.**
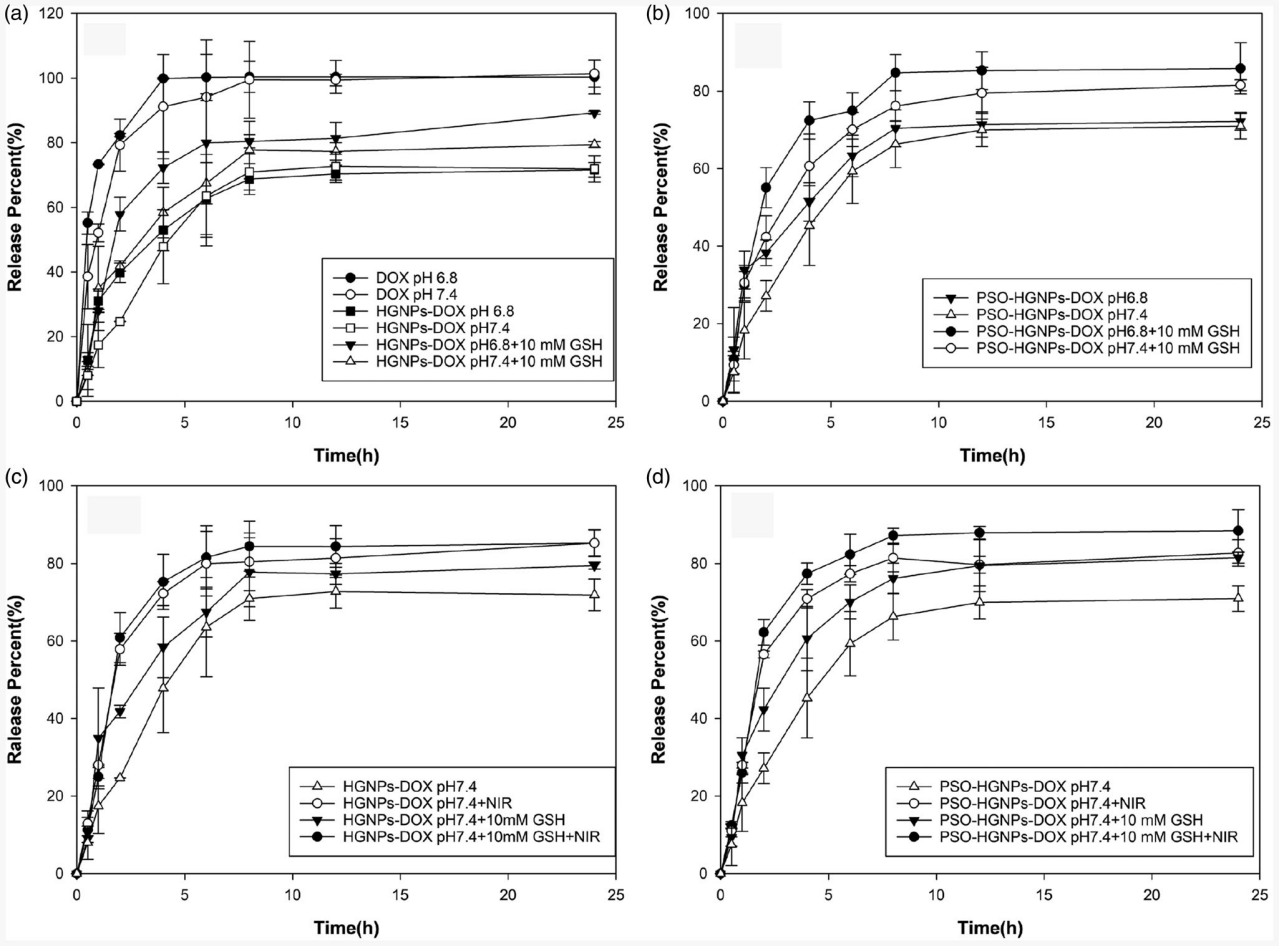
Drug release of free DOX, HGNPs-DOX, and HGNPs-DOX + GSH at pH 6.8 or pH 7.4 (a); Drug release of PSO-HGNPs-DOX and PSO-HGNPs-DOX + GSH at pH 6.8 or pH 7.4 (b). The drug release of HGNPs-DOX and PSO-HGNPs-DOX in the presence or absence of laser irradiation or GSH at pH 7.4 (c,d).

The DOX loading and DOX release from HGNPs-DOX and PSO-HGNPs-DOX under various conditions were evaluated next. As can be seen from Figure 5(a), free DOX was completely released after 6 h and the CRP almost reached 100%. But in the first 2 h, the CRP of free DOX in the acidic environment of pH 6.8 was slightly higher than that in the neutral environment of pH 7.4, which may be due to the greater solubility of DOX in an acidic environment. Moreover, in Figure 5(a), the CRPs of HGNPs-DOX were respectively 52.94% (4 h, pH 6.8) and 47.82% (4 h, pH 7.4), while the CRPs of HGNPs-DOX + 10 mM glutathione (GSH) were respectively 72.25% (4 h, pH 6.8) and 58.37% (4 h, pH 7.4). It was found that the cumulative release of DOX in HGNPs-DOX was less when GSH was not added. It was speculated that DOX was mainly connected to HGNPs through Au-S bonds and the chemical bond was relatively stable, as a result, the drug was not easily released from the medium. However, GSH can promote the release of DOX due to its free sulfhydryl groups, which have a stronger affinity with HGNPs and replace the DOX. Therefore, the DOX cumulative release can be increased with GSH and the drug can be completely released within 24 h. From Figure 5(b), it can be seen that the CRP of PSO-HGNPs-DOX + 10 mM GSH at pH 6.8 and pH 7.4 within 4 h was 72.37% and 60.63%. This was basically consistent with the CRP of HGNPs-DOX + 10 mM GSH within 4 h in Figure 5(a) at 72.25% and 58.37% at pH 6.8 and pH 7.4, respectively. In addition, in the absence of GSH, the CRPs of PSO-HGNPs-DOX and HGNPs-DOX at pH 6.8 and pH 7.4 at each time point possessed no significant difference. Thus, here, it can be proved that the release of the drug would not be affected by the modification of PSO in AuNPs. The release behavior of PSO-HGNPs-DOX and HGNPs-DOX was consistent at different release pH values and in different media.

Then, we investigated the release behavior of DOX from HGNPs under laser irradiation. It can be seen from Figure 5(c), at pH 7.4 that the CRP of HGNPs-DOX was only 47.82% (4 h) and 72.75% (12 h) without NIR irradiation. However, after NIR irradiation, the CRP reached 58.38% (4 h) and 81.03% (12 h). This showed that the release of DOX after NIR laser irradiation increased, which may be due to the enhancement of molecular thermal movement caused by the increase of temperature. It can also be seen in Figure 5(c) that the CRP of HGNPs-DOX + 10 mM GSH + NIR at pH 7.4 reached 75.25% (4 h) and 85.25% (12 h) which was higher than that of the HGNPs-DOX and HGNPs-DOX + 10 mM GSH groups. This indicated that there was a synergistic effect on promoting drug release with GSH and NIR. Furthermore, as can be seen from Figure 5(d), the CRPs from PSO-HGNPs-DOX + 10 mM GSH + NIR at pH 7.4 were 77.36% (4 h) and 87.91% (12 h). There was no significant difference in the cumulative release of PSO-HGNPs-DOX and HGNPs-DOX at each point with or without NIR laser irradiation, indicating that PSO did not change some of the basic properties of the original AuNPs.

In conclusion, these results indicate that the acidic release medium had a positive effect of accelerating the release of DOX. Furthermore, after the addition of GSH or the irradiation by NIR, the release of the drug accelerated significantly, and when HGNPs-DOX underwent both a reduction of GSH and irradiation of NIR, the release of DOX was much faster. In addition, the release behavior of PSO-modified AuNPs was basically consistent with HGNPs-DOX, which proved that PSO did not affect the relevant properties of the AuNPs.

### 3.6. The cytotoxicity of PSO-HGNPs-DOX for triple combination therapy

**Figure 6.**
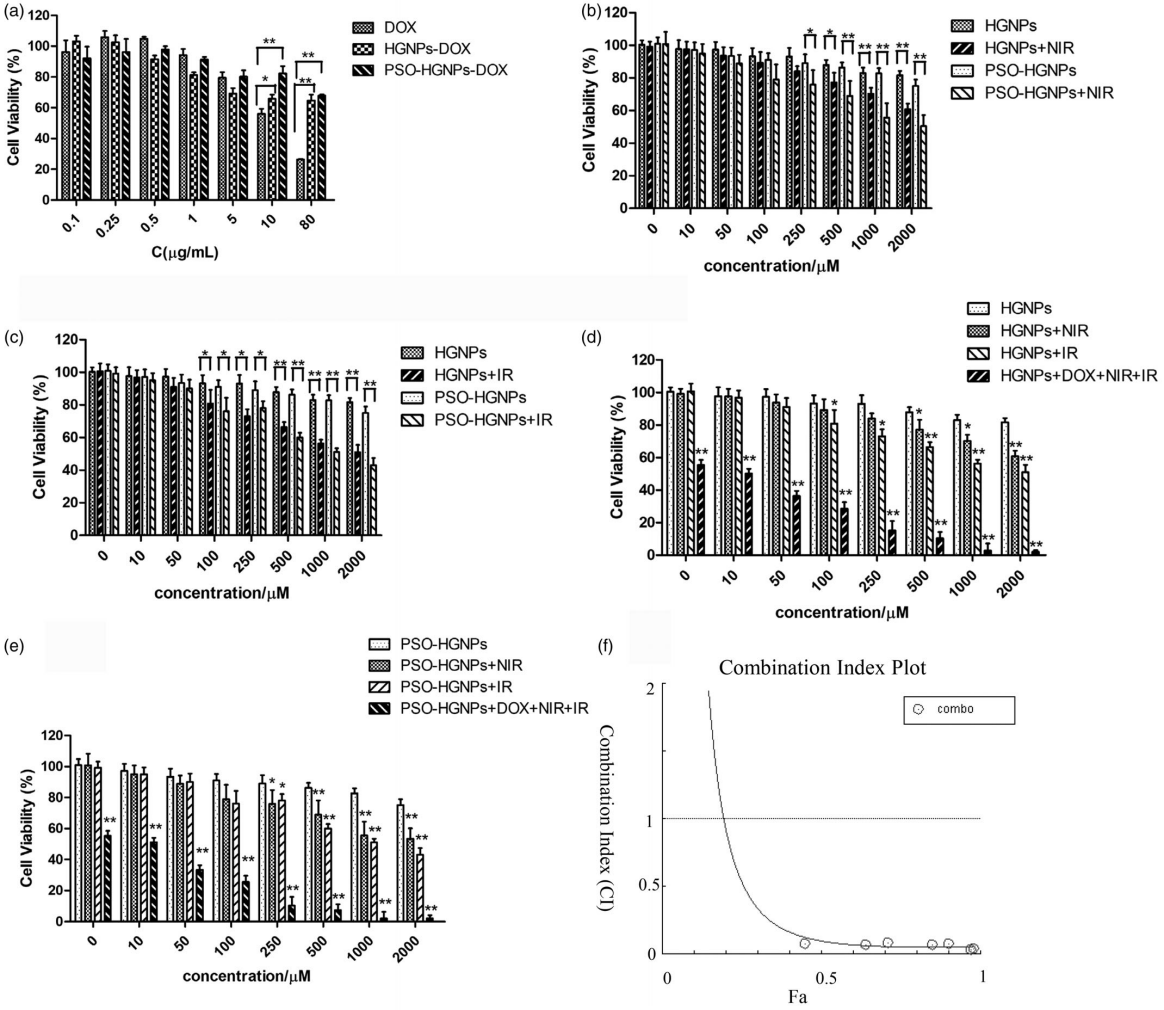
(a) Cell viability of HGNPs, HGNPs-DOX, and PSO-HGNPs-DOX. (b) Cell viability of HGNPs and PSO-HGNPs with NIR. (c) Cell viability of HGNPs with X-ray irradiation (*p < .05, **p < .01). (d) Cell survival of HGNPs and (e) PSO-HGNPs after triple combination therapy (*p < .05, **p < .01, compared with HGNPs or PSO-HGNPs). (f) Combination Index Plot of triple combination therapy.

The results of A549 cell viability after chemotherapy are shown in Figure 6(a). It can be seen that DOX was highly cytotoxic to A549 cells with an IC50 of 8.355 μg/mL. At the highest dose concentration (80 μg/mL, calculated as DOX), the survival rate of cells was 26.33% after free DOX acted on the cells, while the cell survival rates were respectively 64.71% and 67.83% after administration of HGNPs-DOX and PSO-HGNPs-DOX, and were 2.45 times and 2.57 times that of free DOX, respectively. This indicated that when DOX was encapsulated in HGNPs, it effectively reduced the cytotoxicity of the drug to cells and improved the safety of the carrier. Although PSO can promote the uptake of HGNPs-DOX in cells, it can be seen from the results of the stability test in the Supporting Information (Figure S1 ) that PSO-HGNPs-DOX has better serum stability than HGNPs-DOX. Therefore, under the condition of no external stimuli such as NIR or IR, the drug in PSO-HGNPs-DOX was not easy to leak and was less cytotoxic than HGNPs-DOX.

The results of A549 cell viability after thermotherapy is shown in Figure 6(b). It can be seen that for the low concentration administration range (0–250 μM), cell viability of HGNP and HGNPs-PSO was above 90% in the presence or absence of NIR laser irradiation, indicating that low concentrations of HGNP and HGNPs-PSO are safe under laser irradiation. However, for the high concentration range (500–2000 μM), there was a significant difference in cell survival rates between the irradiated and non-irradiated groups of HGNP and PSO-HGNPs. For example, cell viabilities for the HGNP and PSO-HGNP groups without NIR illumination were 81.68% and 78.94%, while under NIR illumination were 60.79% and 50.43%, respectively, at 2000 μM. It was concluded that the higher the concentration of HGNPs, the higher the photothermal conversion efficiency, and the better the therapeutic effect on tumor cells. In addition, at the highest concentration of 2000 μM, PSO-HGNPs had a little bit of a smaller cell survival rate than that for HGNPs. It was speculated that HGNPs modified by PSO can promote the uptake of HGNPs by A549 cells, so the cytotoxicity is greater and the effect of radiotherapy better.

The results of A549 cells after radiotherapy are shown in Figure 6(c). It can be seen that in the concentration range of 0-50 μM (calculated as Au), there was no significant difference in cell viability with or without IR irradiation, but in the concentration range of 100–2000 μM, the cell viability was significantly reduced by IR irradiation. When the concentration of HGNPs was 1000 μM, cell viability for the HGNPs + IR group was only 60%. In addition, HGNPs modified by PSO can promote the uptake of HGNPs by A549 cells, so the cytotoxicity was greater and the effect of radiotherapy better.

Lastly, the cytotoxicity of PSO-HGNPs-DOX for triple combination therapy was investigated. It can be seen from the Figure 6(d,e) that HGNPs and PSO-HGNPs possessed less toxicity to A549 cells in the concentration range of 0–2000 μM, and cell viability was above 85%. However, in the concentration range of 100–2000 μM, when triple combination therapy was used, there was a significant decrease (*p* < .01) in cell viability compared with cells treated with a single treatment. The cell inhibition rate for the PSO-HGNP + DOX + IR + NIR treatment was 97.99% at a concentration of 2000 μM. Compared with the inhibition rate of a single chemotherapy (18.32%), radiotherapy (39.21%), and thermotherapy (49.09%), the inhibition rate of the triple therapy (PSO-HGNP + DOX + IR + NIR) was 5.34, 2.50, and 1.99 times that of the other three monotherapies, respectively, suggesting that they have a good synergistic effect.

Meanwhile, CompuSyn software (ComboSyn, Inc., USA; Abdel-Fattah, 2002; Chou & Martin, 2007) was used to analyze the relationship between the synergy index (CI) and inhibition rate of the triple therapy. When 0.2 < CI < 0.6, the combination therapy had good synergy; when 0.6 < CI < 0.9, the combination therapy had moderate synergy; when 0.9 < CI < 1.1, the combination therapy had only a simple additive effect, but no effective synergy. As shown in the Figure 6(f) when the inhibition rate reached 20%, the CI value of PSO-HGNP + DOX + IR + NIR reached 0.916, indicating that when the inhibition rate was in the range of 20% to 100%, the efficacy of triple therapy was greater than monotherapy. Further, when the DOX concentration in PSO-HGNPs-DOX reached the IC_50_ value (8.3 μg/mL), the CIs were only 0.09, indicating a strengthening of the synergy among hyperthermia, radiotherapy, and chemotherapy.

In conclusion, the triple therapy had the best anti-tumor effect and there was a significant synergy between the three single treatments (**p < .01).

### 3.7. The mechanism of radiotherapy and hyperthermia

**Figure 7.**
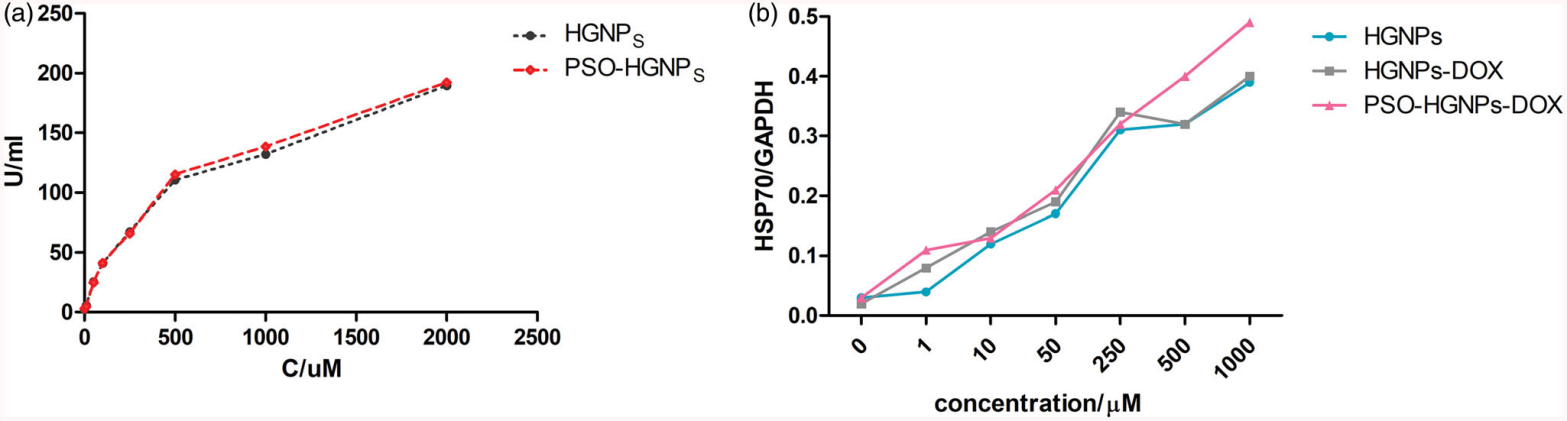
(a) Hydroxylradical-generating ability detection of HGNPs and PSO-HGNPs at different concentrations; (b) Gray analysis of PSO-HGNPs-DOX, HGNPs-DOX, and HGNPs with NIR.

Hydroxyl-radical-generating ability of HGNPs and PSO-HGNPs at different concentrations was detected in Figure 7(a). The hydroxyl-radical-generating ability of HGNPs and PSO-HGNPs rose following an increase in the concentration of HGNPs. However, there was no significant difference in the hydroxyl-radical-generating ability of HGNPs and PSO-HGNPs solutions containing the same gold concentration. It is showed AuNPs can deposit more ion rays at the local tumor site, which can generate more hydroxyl radicals, thereby accelerating the damage of DNA double-strand of tumor cells. It was also proved that AuNPs have a radiosensitivity effect, and the ability of radiosensitization is related to the content of AuNPs.

It can be seen from Figure 7(b) that HSP70 was hardly expressed when the concentration of HGNPs was 0 μM. However, with an increase in concentration, the ratio of HSP70/GAPDH increased, indicating that the HSP70 produced by NIR irradiation in A549 cells increased. When a high concentration of HGNPs (1000 μM) was added to A549 cells, the HSP70/GAPDH ratio was 0.39 after thermotherapy, which was 13 times higher than that without HGNPs for thermotherapy. At the same time, HSP70 produced by a high-concentration HGNPs was 3.25 times higher than that of a low-concentration of HGNPs (10 μM). Here, it was proved that with an increase of HGNPs, HSP70 produced by cells increased significantly after thermotherapy, and there was a significant difference (*p* < .01) between HSP70 produced by the high-concentration HGNPs and low-concentration HGNPs.

In addition, the HSP70/GAPDH ratio of PSO-HGNPs-DOX solutions was 0.49, which was higher than that of HGNPs-DOX (0.4) and HGNPs (0.39; the concentration of Au was 1000 μM). It was shown that PSO-HGNPs taken up by A549 cells was greater than that of HGNPs due to LDL-R mediated endocytosis. Therefore, PSO-HGNPs under NIR irradiation have a higher efficiency for photothermal conversion, causing a HSP70/GAPDH ratio of PSO-HGNPs relatively higher than that of HGNPs-DOX and HGNPs.

### 3.8. Intracellular uptake assay

Detection of LDL-R gene expression in different cells was showed in the Supporting Information in Table S1 and Figure S2, it indicated that the LDL-R gene expression in L02 cells was relatively minimal, while the LDL-R gene expression in A549 cells was the highest and it can be used as the LDL-R gene over-expressing cell line.

**Figure 8.**
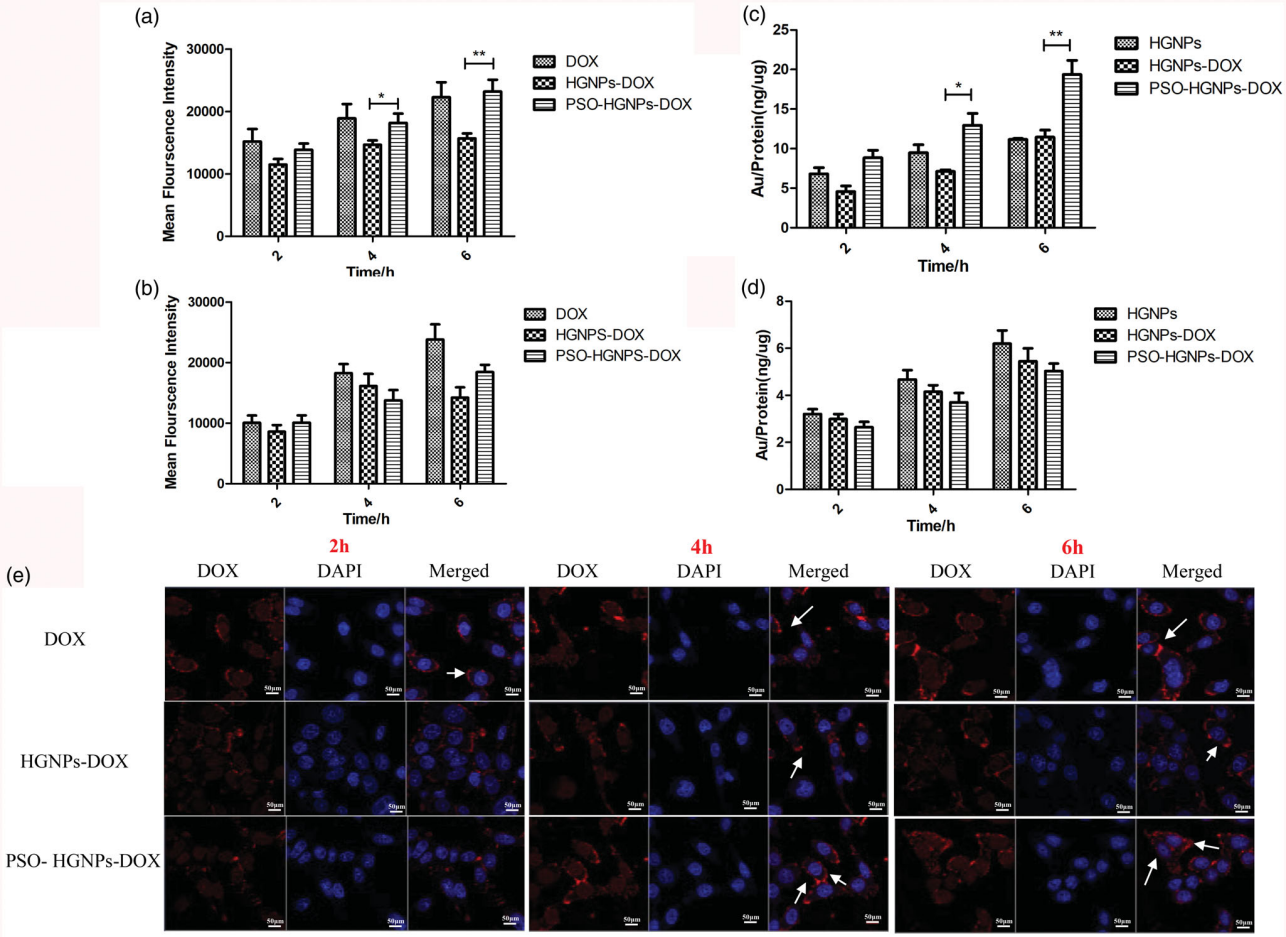
(a) Mean fluorescence intensity of DOX, HGNPs-DOX, and PSO-HGNPs-DOX in A549 and (b) L02 cells. (c)The ratio of Au/protein(ng/μg) of HGNPs, HGNPs-DOX, and PSO-HGNPs-DOX in A549 and (d) L02 cells in intracellular uptake experiments (*p < .05, **p < .01). (e) A549 cell uptake of HGNPs, HGNPs-DOX, and PSO-HGNPs-DOX. Cells were stained with DAPI as red represents the fluorescence of DOX and the white arrows indicate fusion of the drug and cells. (Scale bar = 10 μm).

In Figure 8(a,b), the flow cytometry results of DOX fluorescence intensity for each test group is shown and demonstrated that the fluorescence intensity of PSO-HGNPs-DOX group was higher than the HGNPs-DOX group for A549 cells which overexpress LDL-R. The fluorescence intensity of PSO-HGNP-DOX was 1.48 fold higher for the HGNP-DOX group at 8 h (*p* < .01). Meanwhile, there was no significant difference in the fluorescence intensity between the HGNPs-DOX and PSO-HGNPs-DOX groups for the L02 cells with a low expression of LDL-R. In addition, it can be seen that the uptake of free DOX by the two cells was the highest, presumably due to DOX loading onto the surface of HGNPs, causing the fluorescence of a portion of DOX to quench. Thus, the fluorescence values ​measured for the HGNPs-DOX and PSO-HGNPs-DOX groups were smaller than the amount of DOX taken up in the actual cells. In view of this situation, it was intended to measure the content of Au in the carrier taken up in the cell by AAS, and determine protein content in the cells with the BCA kit. Then, the Au/protein ratio was calculated to quantitatively determine the amount of gold in the cells.

Cell uptake kinetics for the HGNPs, HGNPs-DOX, and PSO-HGNPs-DOX solutions after 2, 4, and 6 h are shown in Figure 8(c,d). The uptake of HGNPs, HGNPs-DOX, and PSO-HGNPs-DOX in A549 cells and L02 cells was time-dependent, and the ratio of Au/protein increased with time. For L02 cells, the uptake rate of PSO-HGNPs was slightly slower than that for the HGNPs, and there was no significant difference between them. However, for the A549 cells, the uptake of the PSO-HGNPs-DOX group was the fastest, and the ratio of Au/protein was 1.68 fold higher for the HGNP-DOX group at 6 h (*p* < .01). Thus, it is indicated that the uptake rate of HGNPs, HGNPs-DOX, and PSO-HGNPs-DOX in the normal cells was basically the same, but in the tumor cells, the PSO-HGNPs-DOX group had a unique advantage of being taken up by the cells in a large amount. Confirming that HGNPs modified by PSO had good targeting ability and could efficiently target overexpressed LDL-R on the surface of A549 cells, thereby increasing the uptake rate.

It can be seen from Figure 8(d) that the fluorescence intensity of the free DOX, HGNPs-DOX, and PSO-HGNPs-DOX group increased with time for 2–6 h, and reached a peak at 6 h. The fluorescence intensity of PSO-HGNPs-DOX was stronger than that of HGNPs-DOX, indicating that the uptake of PSO-HGNPs-DOX by A549 cells was the highest. It has also been qualitatively proven that the HGNPs modified by PSO have good targeting ability. PSO does not affect the photothermal conversion effect, stability, *in vitro* release, and cytotoxicity of HGNPs, but it has a highly effective immune targeting effect, reflecting the superiority of the designed drug carrier (PSO-HGNPs). The fluorescence results were consistent with the results of the cell uptake kinetics experiment.

### 3.9. LDL-R-mediated endocytosis

**Figure 9.**
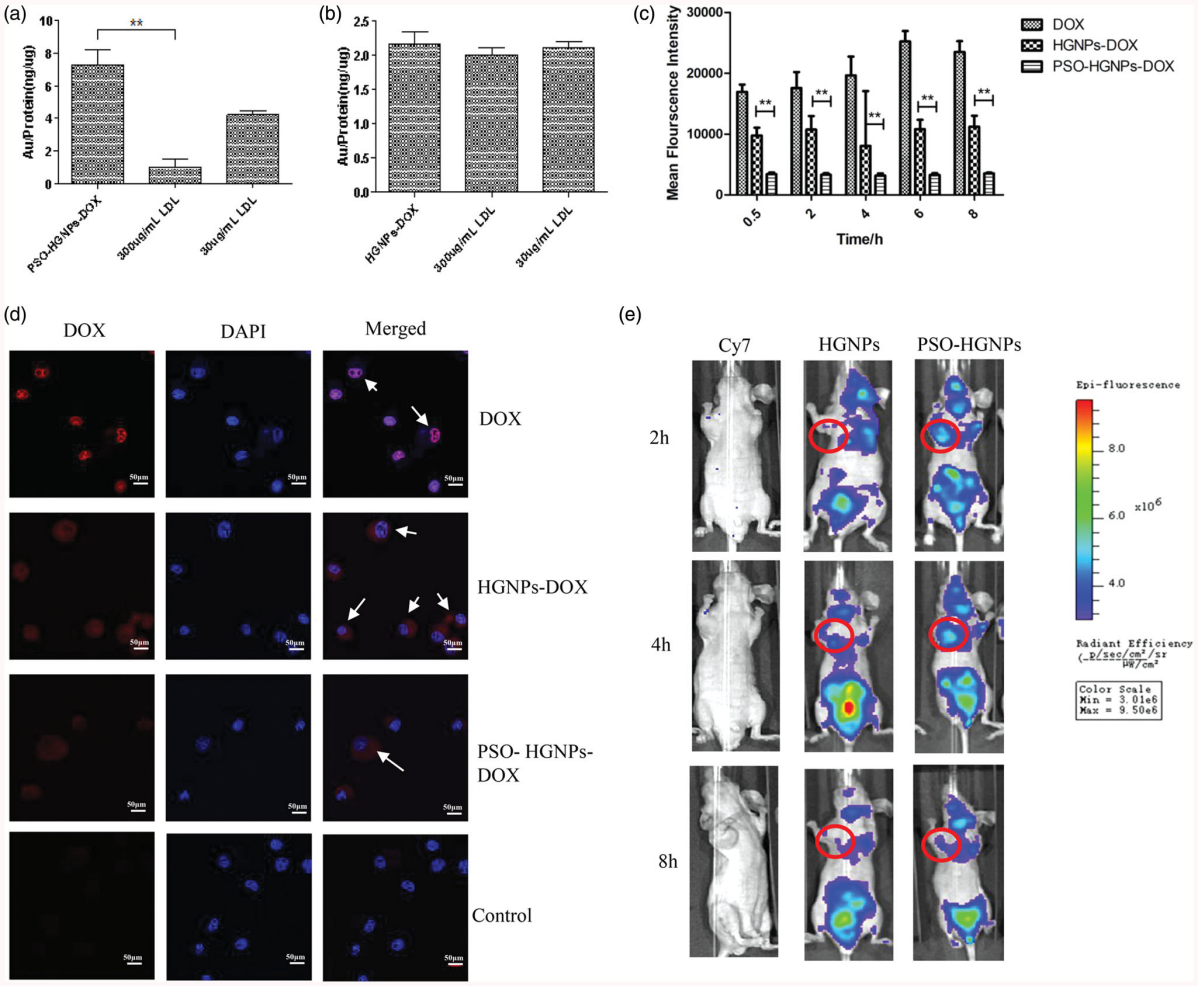
(a) The ratio of Au/protein (ng/μg) of DOX-HGNPs and PSO-HGNPs-DOX in A549 cells. (b) L02 cells in competitive inhibition of LDL-R experiments. (c) Mean fluorescence intensity of HGNPs, HGNPs-DOX, and PSO-HGNPs-DOX (**p < .01). (d) Confocal image of NR8383 cell uptake of HGNPs, HGNPs-DOX, and PSO-HGNPs-DOX at 4 h. Red represents the fluorescence of DOX and blue is the fluorescence of DAPI. White arrows indicate fusion of the drug and cell. (Scale bar = 10 μm). (e) *In vivo* imaging of tumor-bearing mice after administration of Cy7, Cy7-HGNPs, and Cy7-HGNPs-PSO.

It can be seen from Figure 9(a,b) that when LDL was used as a competitive inhibitor, the Au content of the PSO-HGNPs-DOX group was significantly reduced, while the Au content of the HGNPs-DOX group did not significantly change. Moreover, when a high concentration of LDL (300 μg/mL) was used as a competitive inhibitor, the Au content was significantly (**p < .01) lower than that of using a low concentration of LDL (30 μg/mL). It can be inferred that when the LDL concentration was high, there were many receptors bound to LDL on the cells and the amount of HGNPs taken into the cells was reduced, resulting in a lower Au content. Conversely, when a low concentration of LDL was used as a competitive inhibitor, the receptors bound to LDL on the cell was reduced, and the uptake of HGNPs into the cell increases, resulting in an increase in the Au content. The target of PSO-HGNPs-DOX entry into A549 cells is LDL-R on the surface of A549 cells.

### 3.10. Escape macrophage phagocytosis

Drugs themselves are exogenous substances for the body which macrophages may recognize and phagocytose, resulting in a decrease in the efficacy of the drug. The ability of PSO modified HGNPs to escape from lung macrophages was verified by measuring the uptake of rat alveolar macrophage NR8383 cells at different time points. The uptake of different preparations by NR8383 at different time points was measured by flow cytometry (Figure 9(c)), while at the same time, confocal microscopy was used to observe the fluorescence distribution of PSO-HGNPs-DOX (Figure 9(d)).

The results from flow cytometry showed that the amount of DOX uptake was greater than that for the HGNPs and PSO-HGNPs-DOX groups. The uptake of the free DOX and HGNPs-DOX groups increased with time, reaching a maximum after 6 h. However, the fluorescence intensity of PSO-HGNPs-DOX did not change at different times, and the fluorescence intensity of PSO-HGNPs-DOX was one third of that for HGNPs-DOX, and there was a significant difference in uptake for them (**p < .01), which fully illustrated that PSO-HGNPs-DOX has the ability to escape macrophages compared to HGNPs-DOX. In addition, in Figure 9(d), the fluorescence intensity of DOX, HGNPs-DOX, PSO-HGNPs-DOX, and the control group (PBS) entering the cell after 4 h was determined. It can be seen that free DOX had a strong red fluorescence, and the fluorescence intensity merged to the nucleus was also the highest, indicating that free DOX can be taken up into NR8383 cells during 4 h, and had no ability to escape macrophages. The red fluorescence of HGNPs-DOX was slightly weakened compared with the red fluorescence of free DOX, but the content of HGNPs-DOX taken up by macrophages within 4 h was still high due to the absence of their ability to escape macrophages.

Compared with HGNPs-DOX, only a weak red fluorescence of the PSO-HGNPs-DOX group was observed, indicating that the content of PSO-HGNPs-DOX taken up into macrophages was the lowest, proving that PSO-HGNPs-DOX had a good ability to escape macrophages. The above results showed that PSO-HGNPs-DOX was not easily taken up by NR8383 cells, indicating that the modification of PSO increased macrophage escape of HGNPs indeed. It is known that insoluble particles are more easily taken up by macrophages, possibly because they can be enveloped by the lung surfactant protein (SP), which leads to recognition by macrophage antigens (Arredouani et al., 2005, 2006). PSO had greater water solubility, and the modification of PSO could increase the water solubility of AuNPs and prevent them from being endocytosed by macrophages. Therefore, PSO-modified HGNPs can exhibit a better macrophage escape ability and higher drug accumulation in lung tumor to improve a triple combination therapy.

### 3.11. *In vivo* targeting

In this study, tumor-bearing mice were injected with free Cy7, Cy7-HGNPs, and Cy7-HGNPs-PSO through the tail vein, respectively. The *in vivo* distribution of the preparations in A549 tumor-bearing mice was investigated by an IVIS Spectrum *In vivo* Imaging System at 2, 4, and 8 h after administration. As can be seen from Figure 9(e), the free Cy7 group showed no obvious tumor targeting distribution within 8 h after administration. While HGNPs exhibit a tumor-targeted fluorescence distribution, and the fluorescence of the tumor site increased with time, suggesting that HGNPs accumulated in the tumor site. In the PSO-HGNPs group, due to the targeting effect of PSO on the LDL receptors at the tumor site after adsorption of related proteins, the fluorescence intensity at the tumor site after the administration of the preparation was stronger than that of the HGNPs group, and the fluorescence intensity reached its maximum at 4 h. At 8 h, the fluorescence of the PSO-HGNPs group weakened and it is hypothesized that the formulation had a limited residence time at the tumor site, which also provided a reference for the optimal time of PTT.

## 4. Conclusions

This study aimed to construct a PSO modified HGNPs drug delivery system that can target LDL receptors which are overexpressed on the surface of lung cancer cells for a novel triple combination therapy to also escape macrophage phagocytosis. In this work, PSO-HGNPs-DOX was constructed and the suitable particle sizes a desired NIR wavelength (especially 800 nm) was obtained. Then, the photothermal conduction efficiencies of PSO-HGNPs-DOX under NIR were investigated. The result showed that the temperature was significantly increased within 10 min, and PSO-HGNPs-DOX had good photothermal properties. Furthermore, we studied the *in vitro* release of the preparations, in which the cumulative release rate of DOX in pH 6.8 was greater than that in pH 7.4. Furthermore, after the addition of GSH or irradiation by NIR, the release of the drug was accelerated significantly and the modification of PSO did not affect DOS release.

In addition, in this experiment, *in vitro* cytotoxicity, the synergistic effect of triple combination therapy, cellular uptake and macrophage escape behavior of PSO-HGNPs-DOX were systematically studied. The results from cytotoxicity experiments showed that tumor cells treated with thermotherapy and radiotherapy alone only showed good cytostatic rates at higher concentrations, while cells treated with a triple combination therapy had a good synergistic effect at almost all concentrations. When the inhibition rate was in the range of 20% to 100%, the CI of the triple therapy was 0.916 (<1.0), indicating that the efficacy of the triple therapy was greater than monotherapy. Moreover, the expression of LDL-R in different cells was determined in this experiment. The results showed that LDL-R was more highly expressed in A549 cells compared with L02 cells. From the cellular uptake kinetic experiments, the uptake of the preparations by A549 cells gradually increased with time, and the uptake of PSO-HGNPs-DOX was significantly higher than that of HGNPs-DOX. The LDL-R competitive inhibition assay confirmed that LDL-R was the target of PSO-HGNPs-DOX entry into A549 cells. Macrophage escape experiments showed that PSO-HGNPs-DOX significantly escaped macrophage determine to improve a therapeutic effect.

Finally, the targeting of PSO-HGNPs-DOX was investigated in animals by small animal live imaging. Results showed that PSO-HGNPs had better targeting than HGNPs and free cy7 *in vivo*. This provides a basis for future research on the pharmacodynamics and pharmacokinetics of the novel nano preparations in animals.

In summary, PSO-modified HGNPs not only involve simple preparation methods and good stability but they also exhibit excellent antitumor effects by achieving synergetic efficacy of chemotherapy and PTT under NIR laser irradiation. This study also shows a good triple therapeutic effect with LDL receptor targeting function and macrophage escape function. This study introduces a new type of drug carrier with triple combination therapy via LDL-R mediated endocytosis for improved lung cancer treatment.

## Supplementary Material

Supplemental Material
