# Supplementary material for: A polyoxyethylene sorbitan oleate modified hollow gold nanoparticle system to escape macrophage phagocytosis designed for triple combination lung cancer therapy via LDL-R mediated endocytosis
Source: Drug Deliv. 2020 Sep 23;27(1):1342–59. doi: 10.1080/10717544.2020.1822459 (PMC7534200; doi:10.1080/10717544.2020.1822459)
Supplement: Supplemental Material [file IDRD_A_1822459_SM3801.docx]

**Polyoxyethylene sorbitan oleate modified hollow gold nanoparticles system to escape macrophage phagocytosis designed for triple combination therapy via LDLR mediated endocytosis**

**Supporting information**

**Methods:**

**The degree of substitution of LA by elemental analysis**

The reaction was carried out at a molar ratio of lipoic acid and PSO of 1.5: 1 and 2: 1, respectively, and the products obtained were labeled as PSO-LA-1 and PSO-LA-2. Appropriate amounts of PSO-LA-1 and PSO-LA-2 were taken respectively, and the mass percentages of C, H, and S elements were determined using elemental analysis methods. The degree of substitution of lipoic acid was calculated according to Formula (1) ^35^:

 (1)

Where R_C / S_: mass ratio of carbon and sulfur; n_a_: number of carbon elements in lipoic acid; n_b_: number of carbon elements in PSO; n_c_: number of sulfur elements in lipoic acid; and d_s_: substitution of lipoic acid degree.

**The degree of substitution of LA by the Ellman assay**

The preparation of PSO-LA was carried out at a molar ratio of lipoic acid to PSO of 2:1. Then, PSO-LA was dissolved in DMSO, DTT was added, and the reaction was protected overnight under nitrogen in the dark. The mixture was dialyzed against a 1 KD dialysis bag for 24 hours and lyophilized to obtain the PSO-SH powder.

The degree of LA substitution was determined by the Ellman reagent method^36^. A linear regression plot was drawn with absorbance (A) at 412nm as the ordinate and thiol concentration (c, mM) as the abscissa. PSO and PSO-SH solutions (2 mg/ml) were respectively prepared with phosphate buffer (pH 8.0). Then, the solutions were added with Ellman's reagent and were placed at room temperature in the dark for 15 minutes. The absorbance of the PSO solution was set as "Blank", and the absorbance of the PSO-SH solution was measured at a wavelength of 412 nm and the content of –SH according to the linear regression equation. The degree of substitution (ds) of lipoic acid was determined by Equation (2):

 (2)

Where Ds: degree of substitution of lipoic acid; c: thiol content calculated from the corresponding standard curve; n: number of sulfur in the formula of lipoic acid; M_LA_: relative molecular mass of lipoic acid; M_PSO_: approximate molecular weight of PSO; and m: the quality of the weighed PSO-SH.

**Synthesis of HGNPs and HGNPs-DOX**

Briefly, 6 mL of 0.05 M aqueous sodium citrate solution, 300μL of aqueous 0.4 M cobalt chloride (CoCl_2_) and 300μL of 20% poly-N-vinylpyrrolidone (PVP K30) were added to 300 mL of ultrapure water in a three-necked round-bottom flask. The clear solution was pumped down for 15 min to extract the air, after that, 3 mL of 0.1 M sodium borohydride (NaBH_4_) was added to the solution and stirred for 15 min, then stop pumping and a total of 900μL of 25 mM chloroauric acid solution was added, and the mixture was stirred in the air and shake the solution violently until the color was completely changed to green to oxidize the remaining inner cobalt nanoparticle cores to obtain a HGNPs solution.

The prepared HGNPs were centrifuged for 20 min at the 10000 rpm using the centrifuge (KDC-140HR, AnHui ustc ZonKia scientific instruments co., LTD). The precipitate was collected and redispersed for further use. An appropriate amount of DOX solution was added to the HGNPs solution, and incubated at 37 ° C for 8 hours. After the incubation, the HGNPs-DOX solution was centrifuged at 10,000 rpm for 15 minutes. The unreacted DOX in the supernatant was discarded and reconstituted with ultrapure water to obtain HGNPs-DOX.

**Stability of PSO-HGNPs-DOX**

HGNPs, HGNPs-DOX and PSO-HGNPs-DOX aqueous solutions were prepared at a concentration of 0.75 mM (calculated as Au), and the particle diameters of the samples were measured at 0, 1, 2, 4, 8, 16 and 30 days, respectively, and the changes of particle size were recorded. At the same time, the HGNPs, HGNPs-DOX and PSO-HGNPs-DOX solutions were dispersed in 5% serum to prepare a solution with a concentration of 0.75 mM . The particle size of the sample solutions was measured at 0, 2, 4, 6, 8, 12 and 24 h, and the change of particle size was recorded as well.

**Cell viability after chemotherapy, thermotherapy and radiotherapy alone on A549 cells**

The cell viability of A549 cells after chemotherapy was mainly investigated by measuring the cytotoxicity of different kinds of solutions on the cells. The A549 cells were treated with DOX, HGNPs-DOX and PSO-HGNPs-DOX solutions with increasing concentrations (calculated as DOX), incubated again for 24 hours. The cell viability of A549 cells after thermotherapy was examined mainly by adding increasing concentrations of HGNPs and PSO-HGNPs solution (calculated as Au) for 12 h. and were irradiated by NIR (5 w/cm^2^, 5 min). The cells were cultured for 4 hours and irradiated with NIR again with the same power and laser irradiation time. Finally, cell viability was evaluated after the cells were cultured for an additional 8 hours. The viability of A549 cells after radiotherapy was observed by adding the prepared HGNPs and PSO-HGNPs solution then irradiating with X-ray radiation(IR) at a dose of 20Gy (Model 120D, Scanray Corporation, USA). After an additional culturing for 12 h, cell viability was evaluated.

A549 cells were treated with the above chemotherapy, thermotherapy and radiotherapy alone. Then, 100μL of MTT (0.5 mg/mL) was added and incubated for 4h at 37 ºC to form formazan crystals. The medium was removed before 150μl of dimethyl sulfoxide (DMSO) was added to each well and the absorbance of the dissolved formazan was measured at 570 nm using a microplate reader (BioTek USA).

**Detection of LDL-R gene expression in different cells**

1 ml of prechilled TRIzol was added to the digested and dispersed cell sample, and repeatedly pipette to a clear solution without particles. The fully lysed cell solution was left at room temperature for 5 minutes, so that the nucleic acid protein complex was completely separated, and then chloroform (0.2 ml of chloroform per 1 ml of TRIzol) was added and shaken by hand for 15 seconds. The solution was milky and left at room temperature 3min. Then，the above sample was centrifuged at 12000g and 4 ºC for 15min. The sample was separated into three layers after centrifugation: a colorless supernatant water phase, a middle white layer and a pink lower organic phase. An equal volume of isopropanol was added to the obtained supernatant water phase, mixed gently and left at room temperature for 10 min. Then the above sample was centrifuged at 12,000 g at 4 ºC for 10 minutes as well. After removing the supernatant, 1 mL of 70% ethanol (prepared with DEPC-treated water) was slowly added along the tube wall and mixed gently. Finally, the sample was centrifuged at 12000g and 4 ºC for 10min. The precipitate was dried at room temperature for about 5min, and 30 ~ 50 μL of RNase-free water was added to dissolve the RNA precipitate. After the RNA was completely dissolved and stored at -70 ºC.

2 μg of RNA (2μg), 2 μL of OligodT (20μM) was added in a sterile, nuclease-free PCR tube in sequence, and diluted to a total volume of 12.5μLwith double-distilled water without nuclease at 65 ° C for 5 min, and then bath on ice for 5 min. 0.5μL of RNase inhibitor (40 μg/μL), 4.0μL of 5×Reaction Buffer, 2.0μL of dNTPs (10 mM), and 1.0μL of M-MuLV to the PCR tube in order. After the above solution was gently mixed, it was centrifuged at 2000 rpm for 20s. Then the samples were incubated at 42 ºC for 1 h, 70 ºC for 10 min and finally kept on ice for 5 min to obtain cDNA. cDNA samples from different cells were diluted 10-fold (5μL cDNA + 45μL H_2_O), then 10μL of 2 X Realltime PCR Master Mix (SYBR Green)，1μL of template (cDNA diluted 10-fold), 2μL of Primer MIX (10μM F / R each) and 0.1% DEPC water were added to the 0.1mL PCR tube in sequence to complete Real time-PCR experiments.

2-ΔΔCt method for data processing was used in the experiment . ΔCt was the Ct value of the LDL-R gene of different cells minus the Ct value of β-actin gene in the cell, that is, ΔCt_x_=ΔCt_x,LDL-R_-ΔCt_x,β-actin_ (x means different cell types). Here we take L02 as a reference, If the gene expression level of L02 cells was 1, then ΔΔCt_x_=ΔCt_x_-ΔCt_L02_. Finally, the relative expression of LDL-R gene in different kinds of cells could be obtained.

**Result**

**Synthesis of HGNPs and HGNPs-DOX**

HGNPs and HGNPs-DOX was Synthesized sucessfully.

**Stability of PSO-HGNPs-DOX**

The stability of the gold nanoparticle solution in aqueous solution was shown in figure.S1(a). Within 30 days, the HGNP particle size increased from 54 nm to 95 nm, the HGNPs-DOX particle size increased from 72 nm to 110 nm, and that of PSO-HGNPs-DOX increased from 100 nm to 110 nm. There was no significant difference in the particle size change of PSO-HGNPs-DOX within 30 days, possibly due to the modification of PSO and the stability of gold nanoparticles improved, which was conducive for the present gold carrier to achieve multifunctional treatment.

As can be seen from the figure.S1(b), the particle size of HGNPs, HGNPs-DOX and PSO-HGNPs-DOX in serum did not change significantly within 24 hours. Thus, it can be concluded that the gold nanoparticles are stable in serum. In addition, there was no obvious aggregation phenomenon for these gold nanoparticles.


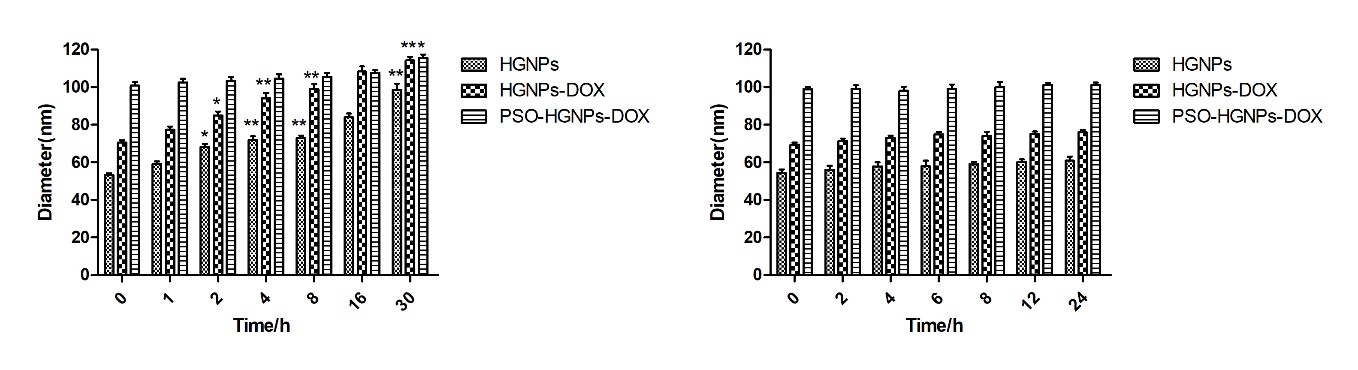


Figure.S1 [Change](file:///E:\硕士三年——科研\沈老师课题\yangershuang\AppData\Local\Youdao\Dict\Application\8.5.1.0\resultui\html\index.html#/javascript:;) of particle size of HGNPs in [aqueous](E:\\硕士三年——科研\\沈老师课题\\yangershuang\\AppData\\Local\\Youdao\\Dict\\Application\\8.5.1.0\\resultui\\html\\index.html#/javascript:;) [solution](file:///E:\硕士三年——科研\沈老师课题\yangershuang\AppData\Local\Youdao\Dict\Application\8.5.1.0\resultui\html\index.html#/javascript:;) (a) and a 5% serum solution (b) (*p<0.05, **p<0.01, comparing with 0 day or 0 h).

**Detection of LDL-R gene expression in different cells**

It can be seen from Figure. S3 (a-b) that the amplification curves of LDL-R and β-actin are S-shaped and relatively smooth. The Ct values of the same gene samples are similar, there is no abnormal curve and the reproducibility is good, indicating the sample quality was better. The melting curves of β-actin gene and LDL-R gene products are shown in Figure. S3 (c-d). Both figureures show a single melting peak, indicating that no primer dimers were formed and no non-specific signals were observed. The peak of the melting curve of LDL-R gene product was 84.84 ° C, and the peak of the melting curve of β-actin gene product was 82.76 ° C.

Then, we compared LDLR gene expression in different cell samples. The expression of LDL-R gene in different cells was calculated by the 2^-ΔΔCt^ method. It can be seen from Table.S1 that the LDL-R gene expression in L02 cells was relatively minimal among the three cells, while the LDL-R gene in A549 cells It had the highest expression and can be used as the LDL-R gene over-expressing cell line.

Table.S1 Expression of LDL-R gene in different cells.

| Cell types | ΔCt | 2^-ΔΔCt^ |
| --- | --- | --- |
| L02 | 12.03±0.18 | 1.01±0.13 |
| U87 | 8.09±0.04 | 15.38±0.43^**^ |
| A549 | 3.36±0.04 | 410.1±12.38^**^ |

Note：**p<0.01, comparing with L02


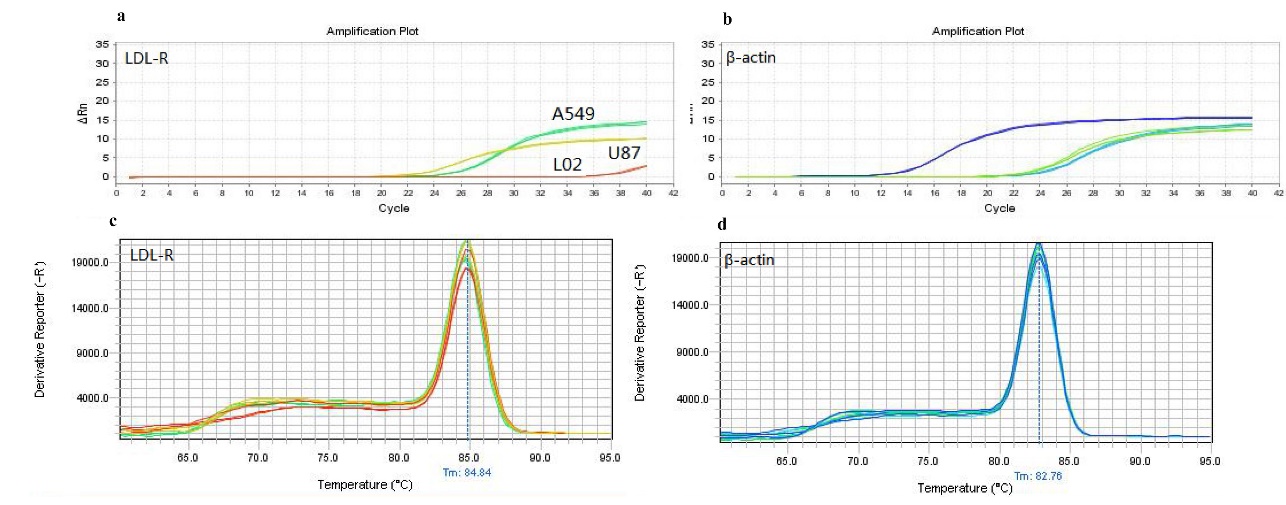


Figure.S3 (a) LDL-R amplification curves in different cells. (b) β-actin amplification curves in different cells. (c) The melting curve of LDL-R in different cells. (d) The melting curve of β-actin in different cells.
